# Introducing the Perfluorinated Cp* Ligand into Coordination Chemistry

**DOI:** 10.1002/anie.202211147

**Published:** 2022-09-21

**Authors:** Robin Sievers, Malte Sellin, Susanne M. Rupf, Joshua Parche, Moritz Malischewski

**Affiliations:** ^1^ Freie Universität Berlin Institut für Chemie und Biochemie, Anorganische Chemie Fabeckstraße 34–36 14195 Berlin Germany; ^2^ Albert-Ludwigs-Universität Freiburg Institut für Anorganische und Analytische Chemie Albertstraße 21 79104 Freiburg Germany

**Keywords:** Cyclopentadienyl Ligands, Density Functional Calculations, Fluorinated Ligands, Rhodium, Substituent Effects

## Abstract

The reaction of AgBF_4_ and [Rh(COD)Cl]_2_ (COD=1,5‐cyclooctadiene) in presence of [NEt_4_][C_5_(CF_3_)_5_] afforded the fluorocarbon soluble complex [Rh(COD)(C_5_(CF_3_)_5_)] by salt metathesis. This complex represents the first example for a successful coordination of the weakly basic [C_5_(CF_3_)_5_]^−^ ligand, since its first synthesis in 1980. In addition to [Rh(COD)(C_5_(CF_3_)_5_)] also the byproduct [Rh(COD)(C_5_(CF_3_)_4_H)] was isolated and fully characterized. Accompanying DFT studies showed that the interaction energy of the [C_5_(CF_3_)_5_]^−^ ligand towards the 12‐electron fragment [Rh(COD)]^+^ is ≈70 kcal mol^−1^ lower in comparison to [C_5_(CH_3_)_5_]^−^ due to reduced electrostatic interactions and weaker π‐donor properties of the ligand. The quantitative but reversible substitution of the [C_5_(CF_3_)_5_]^−^ ligand by toluene, converting it into a weakly coordinating anion, experimentally proved the extraordinary weak bonding interaction.

## Graphical abstract

The perfluorinated Cp* analogue [C_5_(CF_3_)_5_]^−^ resisted all coordination attempts for more than 40 years. Now its first transition metal complex [Rh(COD)(C_5_(CF_3_)_5_)] is reported. The exceptionally weak binding character of [C_5_(CF_3_)_5_]^−^ is proved by DFT calculations and substitution reactions with arenes, allowing to switch between ligand and weakly coordinating anion.

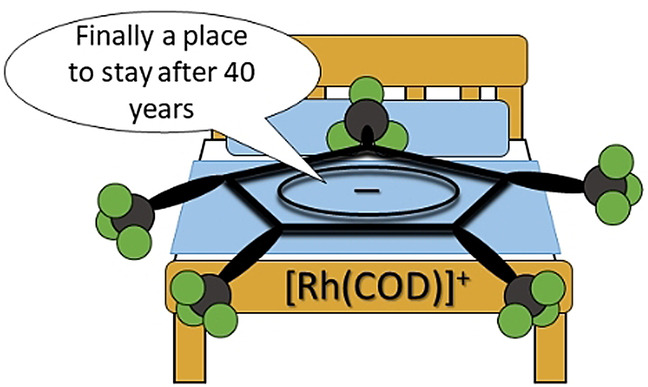

Cyclopentadienyl anions (Cp) are by number the most common organic ligands for transition metals, explaining their ubiquity in organometallic chemistry. Given a variety of Cp derivatives, most of them are electron rich and act as strong π‐donor ligands due to (per)alkylation[^1^, ^2^] or (per)arylation.[^3^, ^4^] Of particular interest is the commonly used [C_5_(CH_3_)_5_]^−^ ligand, better known as Cp*, which finds for example application in highly oxidized transition metal complexes.[^5^, ^6^] Acceptor substituted and electron poor Cp ligands are in contrast less explored, but not of minor interest, as they offer access to electron deficient transition metal complexes with potential application in catalysis. In this context, perfunctionalization through the introduction of carboxy^[7]^ and cyano groups,[^8^, ^9^] or halogenation[^10^, ^11^] is particularly noteworthy. An effective and straightforward approach aims for fluorination, which allows to investigate extraordinary electrophilic and oxidation resistant complexes, that offer fluorocarbon solubility. Beside the direct fluorination of Cp,^[12]^ the introduction of highly fluorinated ponytails,[^13^, ^14^, ^15^] pentafluorophenyl[^16^, ^17^, ^18^] or the discussed trifluoromethyl groups[^19^, ^20^] are feasible strategies.

Depending on the fluorine‐containing substituent, the ligand properties of such Cp derivatives vary. They can be compared by considering the corresponding MO energies (BP86‐D3(BJ)/def2‐TZVP) (Figure 1). By introducing electron withdrawing substituents the frontier orbitals are progressively reduced in energy, resulting in strengthened δ‐acceptor and weakened π‐ and σ‐donor abilities. In comparison to [C_5_H_5_]^−^, [C_5_F_5_]^−^ is still a conceivable ligand due to the strong +M‐effect of fluorine. Contrarily, the perfluorinated Cp* analogue [C_5_(CF_3_)_5_]^−^, reveals significantly lowered MO energies, indicating strongly reduced donor abilities and expectably weaker bonding interactions. Accordingly, the p*K*_a_ values of C_5_H_6_ (p*K*_a_=15.5) and HC_5_F_5_ (estimated p*K*_a_≈13–15) are rather similar, which is in contrast to the high acidity of HC_5_(CF_3_)_5_ (p*K*_a_=−2.2).[^21^, ^22^]

**Figure 1:**
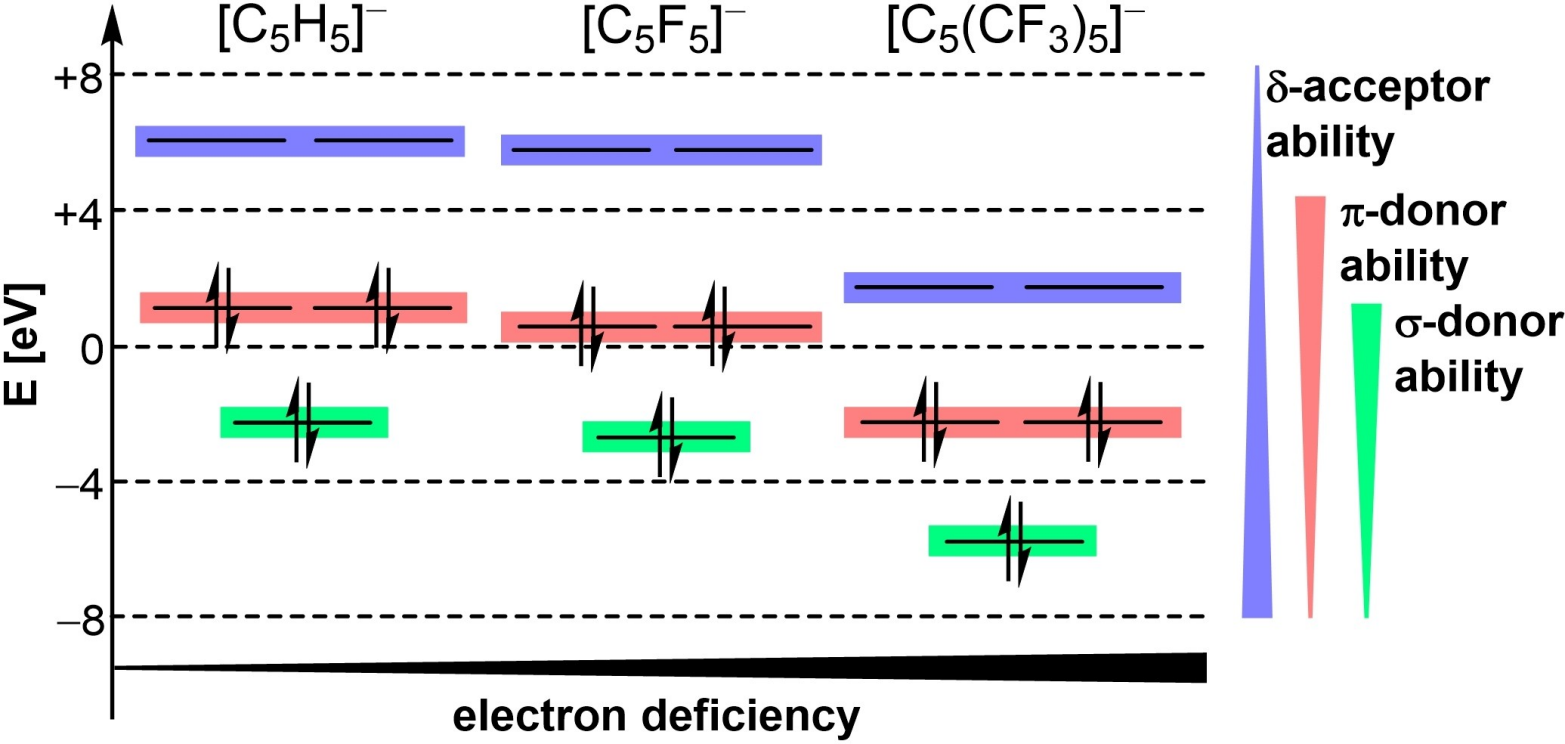
Simplified MO energies in eV and associated donor/acceptor abilities of [C_5_H_5_]^−^, [C_5_F_5_]^−^ and [C_5_(CF_3_)_5_]^−^ (BP86‐D3(BJ)/def2‐TZVP).

Despite the importance of organofluorine compounds, (per)fluorinated Cp ligands are rare in organometallic chemistry due to the remarkable synthetic difficulty of their coordination. [C_5_F_5_]^−^ was first synthesized by Seppelt et al. in 1984 by a challenging multistep synthesis from C_5_Cl_6_.^[21]^ However, various attempts of the coordination to transition metal centers failed, using either the anion or the neutral HC_5_F_5_ due to the insufficient stability towards fluoride eliminations and subsequent polymerization.[^23^, ^24^] The first [C_5_F_5_]^−^ coordination was reported by Hughes et al. in 1992 with a ligand generation in the coordination sphere by flash vacuum pyrolysis to afford [Ru(C_5_(CH_3_)_5_)(C_5_F_5_)].[^25^, ^26^] Subsequently, the group of Sünkel proved access to [Fe(C_5_H_5_)(C_5_F_5_)] by multiple electrophilic fluorinations of lithiated (fluoro)ferrocenes in 2015.^[27]^

The first synthesis of [C_5_(CF_3_)_5_]^−^ was achieved in 1980 by Lemal et al. with a synthetically elaborate, but challenging route starting from a dewarthiophene derivative.^[22]^ In 1995 the group of Chambers presented a simplified access by an improved synthesis from hexachlorobuta‐1,3‐diene and KF in a one‐pot reaction to afford the non‐isolatable K[C_5_(CF_3_)_5_] (scheme 1).[^28^, ^29^] The reaction was improved by the addition of 18‐crown‐6, to increase the solubility of KF.^[30]^ Acidification of the crude material, containing K[C_5_(CF_3_)_5_], with sulfuric acid leads to the generation of HC_5_(CF_3_)_5_. Subsequent reaction with [NEt_4_][OH] gives [NEt_4_][C_5_(CF_3_)_5_] together with minor quantities of [NEt_4_][C_5_(CF_3_)_4_H], which cannot be removed by recrystallization. Although the overall yields are low (5 and 2 %, respectively), amounts of more than 1.0 g are reliably obtained in one batch to offer a stable and storable substrate.

**Scheme 1:**
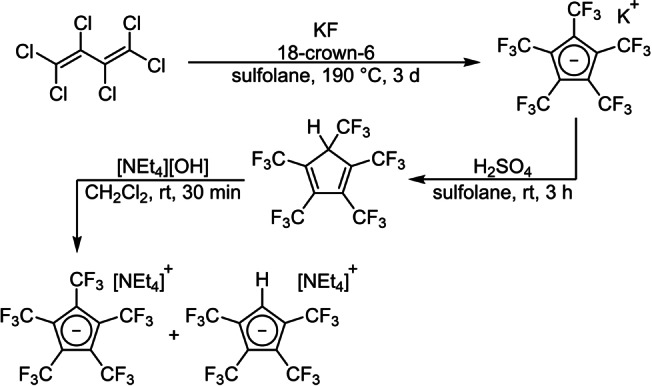
Synthesis of [NEt_4_][C_5_(CF_3_)_5_] and derivative [NEt_4_][C_5_(CF_3_)_4_H] from hexachlorobuta‐1,3‐diene.[^28^, ^29^, ^30^]

Even though the first synthesis of [C_5_(CF_3_)_5_]^−^ was more than four decades ago, all efforts to coordinate this extraordinary electron deficient Cp anion failed. Attempts with transition metal ions in MeCN for example afforded the corresponding solvent complexes with [C_5_(CF_3_)_5_]^−^ counterions, which indicates the pronounced weakly coordinating character of [C_5_(CF_3_)_5_]^−^.^[31]^ Consequently, the aim was to find a synthetically accessible precursor with a sufficient Lewis acidic metal center, to achieve the initial coordination of [C_5_(CF_3_)_5_]^−^. However, very high Lewis acidities were avoided, to suppress fluoride ion abstraction and subsequent decomposition of [C_5_(CF_3_)_5_]^−^. To increase the bonding interaction between metal and Cp ligand, electron rich d^8^‐metals were preferred due to the strengthened backbonding ability into the low lying LUMOs of [C_5_(CF_3_)_5_]^−^. In this context, rhodium was investigated due to its earlier applications in electron deficient and highly fluorinated olefin and allyl complexes.[^32^, ^33^, ^34^, ^35^] Cycloocta‐1,5‐diene (COD) as a co‐ligand allowed to access neutral 18‐electron rhodium(I) complexes, while sufficiently increasing the electron richness of the metal center.

The tetraethylammonium salt (containing [NEt_4_][C_5_(CF_3_)_5_] and [NEt_4_][C_5_(CF_3_)_4_H]) was reacted with the 16‐electron complex [Rh(COD)_2_][BF_4_] (scheme 2, top).^[36]^ While [NEt_4_][C_5_(CF_3_)_5_] was completely unreactive under the given conditions, [NEt_4_][C_5_(CF_3_)_4_H] replaced one COD ligand. Thus, the resulting [Rh(COD)(C_5_(CF_3_)_4_H)] was isolated in 61 % yield, fully characterized and represents the second known coordination of the [C_5_(CF_3_)_4_H]^−^ ligand, since its first coordination in 1992 by Burk et al.^[37]^ The ^19^F NMR spectra revealed the characteristic centrosymmetric multiplet signals at *δ*=−52.6 ppm and −54.8 ppm, with a high field shift in contrast to the ionic [C_5_(CF_3_)_4_H]^−^ (see Supporting Information). In comparison to the electron rich [Rh(COD)(C_5_H_5_)], all ^1^H NMR signals are significantly shifted to lower fields. The remaining hydrogen Cp signal arises at *δ*=5.84 ppm, while the olefinic COD hydrogens are found at *δ*=4.51 ppm.^[38]^ The same applies to the low field shifted COD carbon signals which were observed in the ^13^C{^1^H} NMR spectra. In contrast, the [C_5_(CF_3_)_4_H]^−^ signals appeared in the ^13^C{^19^F} NMR spectra, displaying two singlets at *δ*=121.8 ppm and 121.5 ppm for the trifluoromethyl groups, while Cp carbon signals were distinctly splitted due to ^1^H and ^103^Rh coupling.^[39]^

**Scheme 2:**
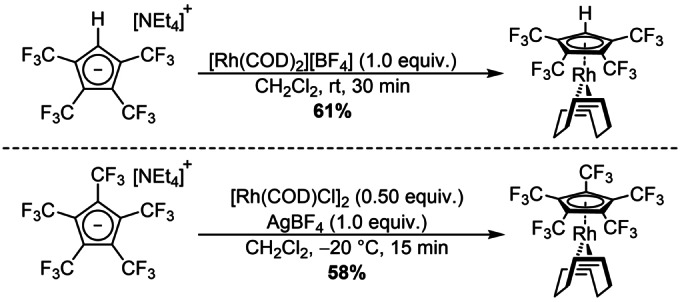
Synthesis of [Rh(COD)(C_5_(CF_3_)_4_H)] (top) and [Rh(COD)(C_5_(CF_3_)_5_)] (bottom).^[36]^

To further increase the Lewis acidity of the metal center, the corresponding [Rh(COD)Cl]_2_ dimer was reacted with AgBF_4_ for an in situ formation of the solvated 12‐electron [Rh(COD)]^+^ fragment in presence of the tetraethylammonium salt (containing [NEt_4_][C_5_(CF_3_)_5_] and [NEt_4_][C_5_(CF_3_)_4_H]) (scheme 2, bottom). While significant decomposition was observed at room temperature, cooling to −20 °C yielded a clean and quantitative conversion. Beside the renewed formation of [Rh(COD)(C_5_(CF_3_)_4_H)] under these conditions, it was possible to isolate [Rh(COD)(C_5_(CF_3_)_5_)] by recrystallization in 58 % yield, which represents the first transition metal complex of this ligand. The ^19^F NMR spectra revealed a singlet at *δ*=−51.1 ppm, that is high field shifted compared to uncoordinated [C_5_(CF_3_)_5_]^−^ (see Supporting Information). The ^1^H NMR spectra showed an even stronger pronounced low field shift for the olefinic COD hydrogens with *δ*=4.68 ppm compared to [Rh(COD)(C_5_(CF_3_)_4_H)] due to the even stronger electron withdrawing character of the perfluorinated ligand. The same trend applies on the COD carbon signals in the ^13^C{^1^H} NMR spectra. Two distinct signals were observed in the ^13^C{^19^F} NMR spectra, giving a singlet at *δ*=121.0 ppm for the trifluoromethyl groups and a doublet due to ^103^Rh coupling at *δ*=96.5 ppm (^1^ *J*_C,Rh_=3.4 Hz) for the Cp carbons.

Both [Rh(COD)(C_5_(CF_3_)_4_H)] and [Rh(COD)(C_5_(CF_3_)_5_)] were obtained as yellow solids that showed unlimited stability at room temperature. Slow decomposition was observed upon contact to air or coordinating solvents.

Single crystals were obtained from solutions in perfluorohexanes or *n*‐pentane, by slowly cooling to −75 °C. [Rh(COD)(C_5_(CF_3_)_4_H)] crystallizes in the monoclinic $P2_{1}/n$ space group (see Supporting Information), while [Rh(COD)(C_5_(CF_3_)_5_)]⋅0.25 *n*‐pentane and unsolvated [Rh(COD)(C_5_(CF_3_)_5_)] (from perfluorohexanes) crystallize in the triclinic space group $P\bar{1}$ (Figure 2, left). Both complexes revealed a η^5^‐coordination of the fluorinated Cp ligands. Interestingly, their Rh‐C_Cp_ bond lengths are only slightly elongated in comparison to [Rh(COD)(C_5_H_5_)] (Table 1).^[38]^ Whereas C_Cp_‐C_Cp_ distances are relatively similar in all three complexes, C=C double bonds of the COD ligand are shortened in [Rh(COD)(C_5_(CF_3_)_4_H)] and [Rh(COD)(C_5_(CF_3_)_5_)], indicating a more electron deficient metal center and consequently less pronounced π‐backbonding to the ligands due to the electron withdrawal of [C_5_(CF_3_)_5_]^−^.

**Figure 2:**
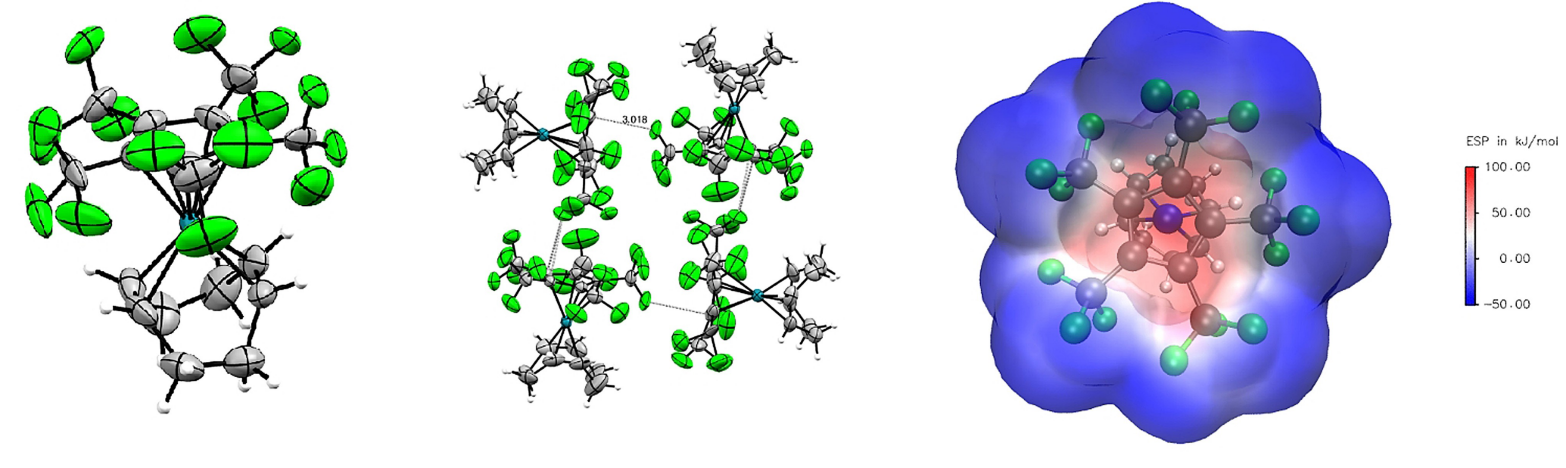
Molecular structure in solid state of [Rh(COD)(C_5_(CF_3_)_5_)]⋅0.25 *n*‐pentane (left), its intermolecular interactions (middle) and the corresponding electrostatic potential surface (right). Disorder and solvent molecules are omitted for clarity. Ellipsoids are depicted with 50 % probability level. Color code: white‐hydrogen, grey‐carbon, green‐fluorine, blue‐rhodium.

**Table 1:** Selected averaged experimental bond lengths in Å of some [Rh(COD)(C_5_R_5_)] complexes.^[a]^

[Rh(COD)(C_5_R_5_)]	Rh−C_Cp_	C_Cp_−C_Cp_	C_COD_=C_COD_
[C_5_H_5_]^−^ ^[38]^	2.255(2)	1.421(2)	1.418(2)
[C_5_(CF_3_)_4_H)]^−^	2.272(3)	1.416(6)	1.403(6)
[C_5_(CF_3_)_5_)]^−^	2.284(4)	1.426(6)	1.404(4)

[a] Bond lengths refer to [Rh(COD)(C_5_(CF_3_)_5_)] (from perfluorohexanes) instead of [Rh(COD)(C_5_(CF_3_)_5_)]⋅0.25 *n*‐pentane.

Furthermore, the molecular structure in solid state revealed the rectangular arrangement of the individual molecules of [Rh(COD)(C_5_(CF_3_)_5_)]⋅0.25 *n*‐pentane. In contrast to the parallel alignment of [Rh(COD)(C_5_(CF_3_)_4_H)] (see Supporting Information), the trifluoromethyl group is pointing directly into the center of the [C_5_(CF_3_)_5_]^−^ moiety of a neighboring molecule (Figure 2, middle). The C⋅⋅⋅F contact distance with 3.018(6) Å is shorter than the sum of the van der Waals radii (3.17 Å),^[40]^ indicating a weak interaction. Apparently, the coordinated [C_5_(CF_3_)_5_]^−^ ligand is sufficiently electron poor and thus partly positively polarized to interact with trifluoromethyl groups,^[41]^ as indicated by the electrostatic potential surface (Figure 2, right).

For further characterization of the novel [C_5_(CF_3_)_5_]^−^ ligand it was aimed to quantify and evaluate its bonding strength in comparison to other Cp ligands,^[42]^ especially the analogous Cp*. Structure optimizations (BP86‐D3(BJ)/def2‐TZVP) were performed for the monocationic, 12‐electron [Rh(COD)]^+^ fragment, several [C_5_R_5_]^−^ ([C_5_(CH_3_)_5_]^−^, [C_5_H_5_]^−^, [C_5_F_5_]^−^, [C_5_(CF_3_)_4_H]^−^, [C_5_(CF_3_)_5_]^−^) and the corresponding neutral, 18‐electron [Rh(COD)(C_5_R_5_)] complexes. From that, the standard enthalpy of reaction Δ*H* ^rxn^ was calculated, which allows to estimate the associated strength of the interaction. Thus, a correlation between the bond strength and the electron richness is revealed, showing [C_5_(CH_3_)_5_]^−^ and [C_5_H_5_]^−^ as the strongest binding Cp ligands with a Δ*H* ^rxn^ of −202 kcal mol^−1^ and −191 kcal mol^−1^, respectively. Even the bond strength of [C_5_F_5_]^−^ is still relatively high with Δ*H* ^rxn^=−173 kcal mol^−1^ due to the strong pronounced +M‐effect of fluorine, that partly compensates its inductive electron withdrawal. By the absence of any conjugative donor effects, the bonding interaction is significantly lowered for [C_5_(CF_3_)_4_H]^−^ with Δ*H* ^rxn^=−138 kcal mol^−1^ and [C_5_(CF_3_)_5_]^−^ with a Δ*H* ^rxn^ of −129 kcal mol^−1^.

For a detailed bonding analysis EDA‐NOCV (BP86‐B3BJ/TZ2P/ZORA) energies were calculated from the structure optimized [Rh(COD)]^+^ fragment and [C_5_R_5_]^−^ ([C_5_(CH_3_)_5_]^−^, [C_5_H_5_]^−^, [C_5_(CF_3_)_5_]^−^, [C_5_(CN)_5_]^−^) (Figure 3 and Table 2). As expected, the interaction energies (*E*_int_) decrease with the electronegativity of the Cp substituents. This mainly derives from the electrostatic interaction energies (*E*_elstat_) between the monocationic [Rh(COD)]^+^ and the monoanionic Cp ligands, since the electron content decreases in the π‐system when electron withdrawing substituents are introduced. Thus, the interaction of [C_5_(CF_3_)_5_]^−^ compared to [C_5_(CH_3_)_5_]^−^ is lowered by Δ*E*_elstat_ *=*63 kcal mol^−1^. For the orbital interaction energies (*E*_orb_) the same trend applies, but herein only a loss of Δ*E*_orb_ *=*43 kcal mol^−1^ from [C_5_(CH_3_)_5_]^−^ to [C_5_(CF_3_)_5_]^−^ is observed. The most important orbital interaction between [Rh(COD)]^+^ and the Cp ligands, the π‐donor ability, is almost halved from *E*_π_=−91 kcal mol^−1^ for [C_5_(CH_3_)_5_]^−^ to *E*_π_=−54 kcal mol^−1^ for [C_5_(CF_3_)_5_]^−^. In comparison, the significantly weaker σ‐donor ability is only slightly decreased in energy from *E*_σ_ *=*−19 kcal mol^−1^ to *E*_σ_ *=*−17 kcal mol^−1^. In contrast to the expectations from the calculated MO energies, the δ‐acceptor ability is only marginally influenced by the perfluorination and only increases from *E*_δ_=−18 kcal mol^−1^ for [C_5_(CH_3_)_5_]^−^ to *E*_δ_=−20 kcal mol^−1^ for [C_5_(CF_3_)_5_]^−^. However, compared to the electron rich [C_5_(CH_3_)_5_]^−^ and [C_5_H_5_]^−^, the δ‐backbonding is more relevant than the σ‐bonding for the overall bonding interaction in [C_5_(CF_3_)_4_H]^−^ and [C_5_(CF_3_)_5_]^−^ and therefore equals a reversed order. Thus, due to minor electrostatic interactions and a strongly decreased π‐donor ability, the extraordinary weak bonding is explained.

**Figure 3:**
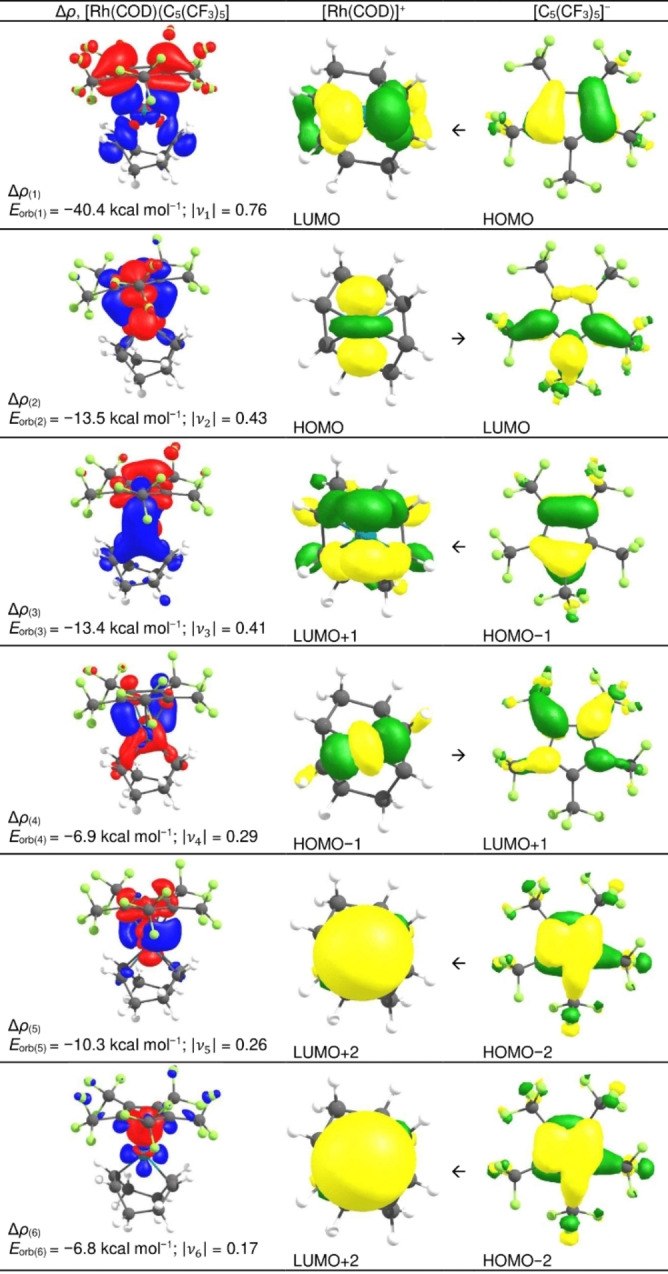
Deformation density plots for [Rh(COD)(C_5_(CF_3_)_5_)] of NOCVs (natural orbitals for chemical valence) 1–6 and most participating SFOs (symmetrized fragment orbitals) with Δ*ρ* (difference in electron density) (BP86*‐*B3BJ/TZ2P/ZORA). Isosurface values=0.05 (SFOs), 0.001 (deformation densities). Charge flow direction in the deformation densities is from red to blue.

**Table 2:** Calculated EDA‐NOCV energies in kcal mol^−1^ for [Rh(COD)(C_5_R_5_)] by variation of the Cp ligand (BP86‐B3BJ/TZ2P/ZORA). Percentage values are related to *E*_orb_.

[Rh(COD)(C_5_R_5_)]	*E*_int_	*E*_Pauli_	*E*_elstat_	*E*_steric_	*E*_disp_	*E*_orb_	NOCV stabilization interaction		
							*E*_σ_ (2×σ‐donor)	*E*_π_ (2×π‐donor)	*E*_δ_ (2×δ‐acceptor)
[C_5_(CH_3_)_5_]^−^	−207	203	−240	−37	−17	−154	−19/**12 %**	−91/**59 %**	−18/**12 %**
[C_5_H_5_]^−^	−193	181	−229	−48	−9	−136	−18/**13 %**	−82/**60 %**	−16/**11 %**
[C_5_F_5_]^−^	−176	201	−224	−23	−10	−144	−10/**7 %**	−83/**58 %**	−32/**22 %**
[C_5_Cl_5_]^−^	−161	183	−199	−16	−16	−129	−22/**17 %**	−68/**53 %**	−18/**14 %**
[C_5_(CF_3_)_4_H]^−^	−145	169	−184	−15	−16	−113	−16/**14 %**	−58/**52 %**	−19/**17 %**
[C_5_(CF_3_)_5_]^−^	−138	168	−177	−9	−18	−111	−17/**15 %**	−54/**48 %**	−20/**18 %**
[C_5_(CN)_5_]^−^^[a]^	−111	149	−145	3	−16	−98	−17/**17 %**	−47/**48 %**	−18/**18 %**

[a] No η^5^‐coordination compound is known for [C_5_(CN)_5_]^−^.

Consequently, an exceptional reactivity is expected for the [C_5_(CF_3_)_5_]^−^ ligand compared to ordinary electron rich Cp ligands. These are commonly introduced by displacing other ligands and thereon mostly show an irreversible binding to transition metal centers.^[43]^ Substitution of Cp ligands by olefins or arenes is only accomplished in presence of other reagents (strong reductants or dihydrogen).[^44^, ^45^] The [C_5_(CF_3_)_5_]^−^ ligand in contrast might offer the possibility of direct substitution by competing ligands and therefore serve as a rare displaceable anionic 6‐electron ligand, enabling access to synthetically valuable solvent complexes for example. In this context, the weak bonding is emphasized, as [Rh(COD)(C_5_(CF_3_)_5_)] was found to undergo an unusual substitution of [C_5_(CF_3_)_5_]^−^ by arenes. In toluene for example, a quantitative displacement was observed within 23 h at room temperature (Figure 4), hereby converting the [C_5_(CF_3_)_5_]^−^ ligand into a WCA (weakly coordinating anion) to yield the isolatable complex [Rh(COD)(PhMe)][C_5_(CF_3_)_5_], as proved by the associated molecular structure in solid state (see Supporting Information).^[46]^ In comparison, when performed in benzene, the reaction struggles to reach full conversion after several days, indicating controllability of the given equilibrium by electronic parameters. Surprisingly, the substitution by arenes appeared to be fully reversible in some solvents, such as chloroform, to release toluene and quantitatively regain [Rh(COD)(C_5_(CF_3_)_5_)] after 27 h at room temperature. As expected, the backreaction appeared to be faster, when performed with the associated benzene complex. Instead, increasingly polar solvents (e.g. dichloromethane) seemed to stabilize the cationic complexes, as conversion was definitely slower.

**Figure 4:**
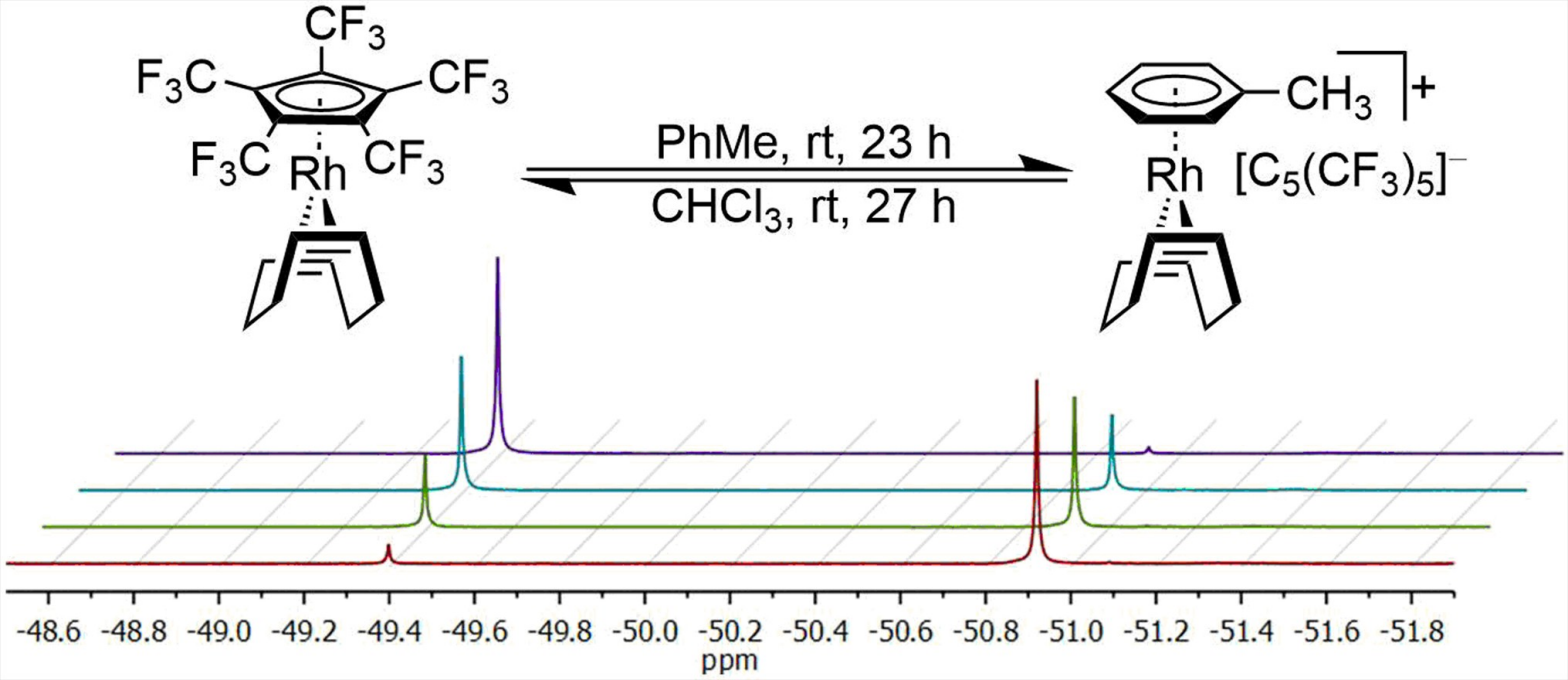
Equilibrium of [Rh(COD)(C_5_(CF_3_)_5_)] (*δ*=−50.9 ppm) and [Rh(COD)(PhMe)][C_5_(CF_3_)_5_] (*δ*=−49.4 ppm) and the ^19^F NMR (377 MHz, rt) spectra of [Rh(COD)(C_5_(CF_3_)_5_)] in *d*_8_‐PhMe after 10 min (red), 1 h (green), 3 h (blue) and 23 h (violet).

In summary, although the coordination of this weakly basic anion has proven challenging since its first synthesis in 1980, with [Rh(COD)(C_5_(CF_3_)_5_)], the first transition metal complex of this ligand is now reported. While its binding motif is similar to electron‐rich Cp ligands, the bonding analysis proved a reduced π‐donor ability when compared to Cp*. This significantly weaker bonding interaction was emphasized by the quantitative and reversible substitution in toluene, allowing to switch [C_5_(CF_3_)_5_]^−^ between ligand and WCA. This unique solvent‐switchable system might offer fascinating future applications not only for coordination chemists but also for fluorous phase‐transfer catalysis.

## Conflict of interest

The authors declare no conflict of interest.

## Supporting information

As a service to our authors and readers, this journal provides supporting information supplied by the authors. Such materials are peer reviewed and may be re‐organized for online delivery, but are not copy‐edited or typeset. Technical support issues arising from supporting information (other than missing files) should be addressed to the authors.

Supporting Information

## Data Availability

The data that support the findings of this study are available in the Supporting Information of this article.
