# Supplementary material for: Introducing the Perfluorinated Cp* Ligand into Coordination Chemistry
Source: Angew Chem Int Ed Engl. 2022 Sep 21;61(43):e202211147. doi: 10.1002/anie.202211147 (PMC9826324; doi:10.1002/anie.202211147)
Supplement: Supplementary file 1 — Supporting Information [file ANIE-61-0-s001.pdf]

Angewandte Chemie
Eine Zeitschrift der Gesellschaft Deutscher Chemiker

Supporting Information

Introducing the Perfluorinated Cp* Ligand into Coordination Chemistry

*R. Sievers, M. Sellin, S. M. Rupf, J. Parche, M. Malischewski**

Contents

General Information.....1
Synthetic Procedures.....4
NMR Spectra.....9
IR Spectra.....21
Crystallographic Data.....23
DFT Calculations.....30
References.....40

General Information

All Reactions and workups (except the recrystallization of $[NEt_4][C_5(CF_3)_4H]$ and $[NEt_4][C_5(CF_3)_5]$ were performed in previously heated glassware under an atmosphere of argon using standard Schlenk techniques and an oil pump vacuum of 10^{-3} mbar. Room temperature (rt) refers to 25 °C. The addition of liquid reagents and solvents was done by using threefold argon-flushed disposable syringes and septa, while solids were added in argon stream. Low temperature reactions were performed in a cooled ethanol-bath. Glassware was cleaned by storing in a potassium hydroxide bath for several days, rinsed with diluted hydrochloric acid and doubly deionized water and dried at 150 °C.

Pressure reactions

The synthesis of $[NEt_4][C_5(CF_3)_5]$ involves high temperatures and highly volatile substances in a closed system. Hence, it must be assumed, that high pressures arise upon heating and advanced caution is required. Therefore, it is advisable to perform the reaction in a separate and properly closed fumehood. The thick-walled glass reaction vessel should not be opened and if possible, not even touched until the reaction has finished and reached rt.

Solvents and reagents

Anhydrous MeCN, CH_2Cl_2, $CHCl_3$, toluene and *n*-pentane were obtained from the solvent system FMBRAUN MB SPS-800 and stored over activated 3 Å mol sieves. Deuterated solvents CD_2Cl_2, $CDCl_3$ and d_8-PhMe were used as purchased and stored over activated 3 Å mol sieves. Solvents were degassed by three freeze-pump-thaw cycles. Sulfolane was heated at 60 °C for at least 24 h over activated 3 Å mol sieves and additionally 1 h in high vacuum prior to use. 18-crown-6 was heated at 80 °C for 2 h in high vacuum prior to use. All other solvents and commercially available reagents were used without further purification.

Nuclear magnetic resonance (NMR) spectroscopy

NMR spectroscopy was measured on a JEOL ECX 400 (400 MHz) or a Varian INOVA 600 (600 MHz) in the reported deuterated solvents $CDCl_3$, CD_2Cl_2 and d_8-PhMe. All given chemical shifts in ^{1}H-NMR spectra are calibrated on the resonance signals of $CHCl_3$ contained in $CDCl_3$ (δ = 7.26 ppm), $CDHCl_2$ contained in CD_2Cl_2 (δ = 5.32 ppm) and d_7-PhMe contained in d_8-PhMe (7.09 ppm, 7.01 ppm, 6.97 ppm and 2.08 ppm). The ^{13}C-NMR spectra are calibrated on the respective resonance signals of $CDCl_3$ (δ = 77.16 ppm), CD_2Cl_2 (δ = 53.84 ppm).[1,2] All other

spectra are device-internal calibrated relative to the resonance signal of tetramethylsilane, by the unified chemical shift scale. The given multiplicities are phenomenological, thus the actual appearance of the signals is stated and not the theoretically expected one. The following abbreviations were used and analogously combined to designate multiplicities: s (singlet), d (doublet), t (triplet), q (quartet), m (multiplet), m_c (centrosymmetric multiplet). For centrosymmetric multiplets the center and for non-symmetric multiplets the interval is stated. Evaluation of spectra was performed with Mestrelab Research MNova 7.[3]

Infrared (IR) spectroscopy

IR spectroscopy was measured on a FT (Fourier transformation) Nicolet iS10 or iS50 IR-spectrometer. The sample was directly measured by ATR (attenuated total reflection) technique. Characteristic absorptions are given in wavenumbers $\tilde{\nu}$ [cm^{-1}] and intensities are stated as vs (very strong), s (strong), m (medium) and w (weak).

High resolution mass spectroscopy (HRMS)

HRMS was recorded using a Varian MAT 711 spectrometer by electron impact ionization (EI) or electrospray ionization (ESI) at the department of mass spectroscopy at the Freie Universität Berlin. A detailed listing of fragmentation is dispensed, instead the molecular ion peak or a characteristic fragment peak is stated.

X-ray diffraction (XRD)

X-Ray data were collected on a BRUKER D8 Venture system. Data were collected at 100(2) K using graphite monochromated Mo K_α radiation (λ_α = 0.71073 Å). The strategy for the data collection was evaluated by using the Smart software. The data were collected by the standard "ψ-ω scan techniques" and were scaled and reduced using Saint+software. The structures were solved by using Olex2,[4] the structure was solved with the XT[5] structure solution program using Intrinsic Phasing and refined with the XL refinement package[6,7] using Least Squares minimization. Part instructions were used to deal with disorder. Bond length and angles were measured with Diamond Crystal and Molecular Structure Visualization Version 4.6.2.[8] Drawings were generated with POV-Ray.[9]

Density functional theory (DFT) calculations

DFT calculations were performed with Gaussian 16.[10] Structure optimizations and population analysis (gas phase) were done using BP86-D3(BJ)/def2-TZVP. EDA-NOCV (gas phase) calculations were done using BP86-B3BJ/TZ2P/ZORA. Solutions were processed using Avogadro[11] and Chemcraft 1.8.[12] Electrostatic surface potentials were calculated with the software MultiWFN[13] and visualized with VMD.[14]

Synthetic Procedures

Tetraethylammonium 1,2,3,4-tetrakis(trifluoromethyl)cyclopentadienide and tetraethylammonium 1,2,3,4,5-pentakis(trifluoromethyl)cyclopentadienide

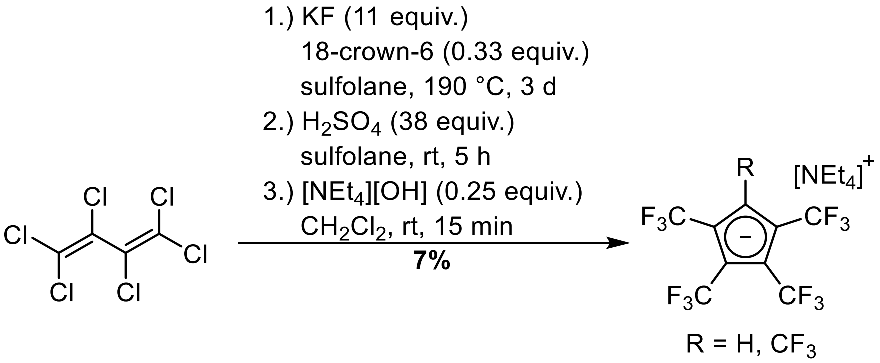

In a dried 1000 mL pressure flask anhydrous KF (60 g, 1.0 mol, 11 equiv.) was placed in anhydrous and degassed sulfolane (190 mL) under an atmosphere of argon. Anhydrous and degassed 18-crown-6 (8.7 g, 33 mmol, 0.33 equiv.) and hexachlorobuta-1,3-diene (15 mL, 96 mmol, 1.0 equiv.) were added at rt. The resulting reaction mixture was carefully shaken and cooled to –196 °C in high vacuum. The properly closed pressure flask was slowly warmed to 190 °C and stirred at this temperature for 3 d. Then the resulting black suspension was cooled to rt and the volatiles were removed in high vacuum. The remaining mixture was filtrated under an atmosphere of argon and the residue was extracted with anhydrous MeCN (3 × 40 mL). The filtrate was warmed to 40 °C and all MeCN was removed in high vacuum, while stirring. The resulting solution was put under high vacuum and H_2SO_4 (conc., 200 mL, 3.6 mol, 38 equiv.) was added dropwise at rt over a period of 3 h, while stirring and continuously collecting the volatiles in a cold trap of –196 °C. After complete addition, the mixture remained for additional 2 h in high vacuum. The cold trap was put under argon and slowly warmed to 0 °C, giving a pale yellow liquid. Then CH_2Cl_2 (20 mL) and a solution of $[NEt_4][OH]$ (35% in water, 10 mL, 24 mmol, 0.25 equiv.) were added and the reaction mixture was stirred for 15 min at rt, giving a deep red solution. The aqueous layer was separated and extracted with CH_2Cl_2 (4 × 20 mL). The combined organic layers were dried over $MgSO_4$, filtrated and the solvent was removed under reduced pressure. The remaining solid was suspended in Et_2O (~5 mL) and recrystallized twice from CH_2Cl_2 (~10 mL) by slowly cooling to –20 °C. The crystalline residue was decanted and washed with Et_2O (2 × 5 mL). The solvents were removed under reduced pressure to give 1.4 g of a colorless crystalline solid, consisting of $[NEt_4][C_5(CF_3)_4H]$ (0.4 g, 0.8 mmol) and $[NEt_4][C_5(CF_3)_5]$ (1.0 g, 1.9 mmol) which cannot be separated.

The molar ratio of $[NEt_4][C_5(CF_3)_4H]$ (30 mol-%) and $[NEt_4][C_5(CF_3)_5]$ (70 mol-%) was calculated by integration of the respective signals in the ^{19}F-NMR spectra, and subsequent division by the number of fluorine atoms. This corresponds to a mass ratio of $[NEt_4][C_5(CF_3)_4H]$ (27 mass-%) and $[NEt_4][C_5(CF_3)_5]$ (73 mass-%) and a average molar mass of 514.83 g/mol. Based on the quantity of hexachlorobuta-1,3-diene (96 mmol) this would correspond to yields of 0.83% for $[NEt_4][C_5(CF_3)_4H]$ and 1.98% for $[NEt_4][C_5(CF_3)_5]$. In order to take the number of carbon atoms into account (9 and 10 in comparison to 4 in the starting material), the yields are multiplied by factors of 9/4 and 10/4 to give 1.87% and 4.95%.

$[NEt_4][C_5(CF_3)_4H]$: ^{1}H-NMR (401 MHz, CD_2Cl_2, rt) δ [ppm] = 6.44 (s, 1H), 2.97 (q, $^3J_{H,H}$ = 7.3 Hz, 8H), 1.20 (t, $^3J_{H,H}$ = 7.3 Hz, 12H). **^{13}C{^{1}H}-NMR** (151 MHz CD_2Cl_2, rt) δ [ppm] = 52.7 (m_c, 4C), 7.3 (s, 4C). **^{19}F-NMR** (377 MHz, CD_2Cl_2, rt) δ [ppm] = –51.3–(–51.4) (m, 6F), –53.9 (m_c, 6F).[15]
$[NEt_4][C_5(CF_3)_5]$: ^{1}H-NMR (401 MHz, CD_2Cl_2, rt) δ [ppm] = 2.97 (q, $^3J_{H,H}$ = 7.3 Hz, 8H), 1.20 (t, $^3J_{H,H}$ = 7.3 Hz, 12H). **^{13}C{^{1}H}-NMR** (151 MHz CD_2Cl_2, rt) δ [ppm] = 52.7 (m_c, 4C), 7.3 (s, 4C). **^{13}C{^{19}F}-NMR** (151 MHz CD_2Cl_2, rt) δ [ppm] = 124.9 (s, 5C), 109.6 (s, 5C). **^{19}F-NMR** (377 MHz, CD_2Cl_2, rt) δ [ppm] = –50.6 (s, 15F).
The analytical data are consistent with those reported in literature.[16,17]

η^2,η^2-(Cycloocta-1,5-diene)-η^5-1,2,3,4-tetrakis(trifluoromethyl)cyclopentadienylrhodium(I)

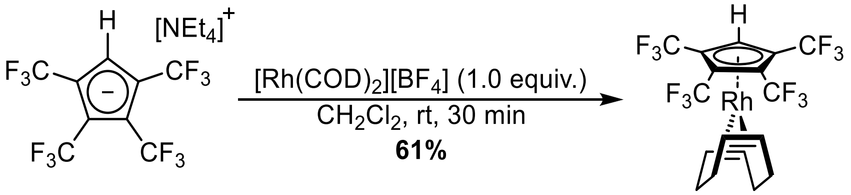

In a dried 10 mL Schlenk flask $[Rh(COD)_2][BF_4]$ (52 mg, 0.13 mmol, 1.0 equiv.) was dissolved in anhydrous CH_2Cl_2 (3 mL) under an atmosphere of argon. A substrate mixture (0.23 g, 0.44 mmol, 30 mol-% $[NEt_4][C_5(CF_3)_4H]$, 70 mol-% $[NEt_4][C_5(CF_3)_5)]$), corresponding to $[NEt_4][C_5(CF_3)_4H]$ (60 mg, 0.13 mmol) was added and the reaction mixture was stirred for 30 min at rt, giving a yellow solution. The solvent was removed in high vacuum and the residue was extracted with perfluorohexanes (3 × 1 mL) and filtrated under an atmosphere of argon. Then the filtrate was concentrated in high vacuum for crystallization by slowly cooling from rt to –75 °C. The crystalline residue was decanted and washed with perfluorohexanes (3 × 0.5 mL), cooled to –78 °C. The

solvent was removed in high vacuum to give product [Rh(COD)($C_5(CF_3)_4H$)] (43 mg, 79 µmol) as bright yellow crystals. Referring to the applied amount of substance of [NEt_4][$C_5(CF_3)_4H$], a yield of 61% is obtained.

^{1}H-NMR (401 MHz, $CDCl_3$, rt) δ [ppm] = 5.84 (s, 1H), 4.51 (s, 4H), 2.35–2.31 (m, 4H), 2.07–2.01 (m, 4H). **^{13}C{^{1}H}-NMR** (151 MHz, $CDCl_3$, rt) δ [ppm] = 74.2 (d, $^1J_{C,Rh}$ = 13.6 Hz, 4C), 31.6 (s, 4C). **^{13}C{^{19}F}-NMR** (151 MHz, $CDCl_3$, rt) δ [ppm] = 121.8 (s, 2C), 121.5 (s, 2C), 97.5 (m, 2C), 94.5 (m, 2C), 88.4 (d, $^1J_{C,H}$ = 184.0 Hz, 1C). **^{19}F-NMR** (377 MHz, $CDCl_3$, rt) δ [ppm] = –52.6 (m_c, 6F), –54.8 (m_c, 6F). **FT-IR** (ATR) ṽ [cm^{-1}] = 598 (s), 698 (s), 733 (m), 757 (m), 801 (m), 821 (m), 865 (m), 880 (m), 945 (s), 1129 (vs), 1223 (vs), 1344 (w), 1431 (m), 1461 (m), 1502 (w), 2843 (w), 2890 (w), 2928 (w), 3139 (w). **HRMS** (EI-TOF, positive) m/z for [$C_{17}H_{13}F_{12}Rh$]$^+$ calculated: 547.9881; measured: 547.9875. **EA** ($C_{17}H_{13}F_{12}Rh$) calculated: C: 37.25%, H: 2.39%; measured: C: 37.76%, H: 2.85%.

η^2,η^2-(Cycloocta-1,5-diene)-η^5-1,2,3,4,5-pentakis(trifluoromethyl)cyclopentadienyl-rhodium(I)

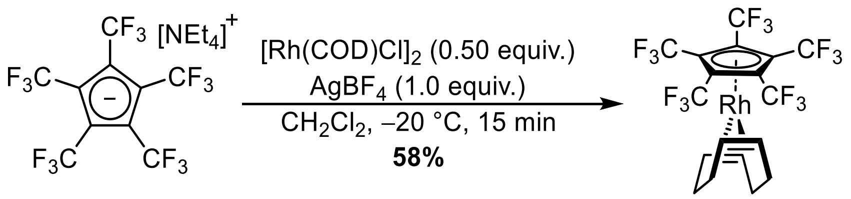

A substrate mixture (0.21 g, 0.41 mmol, 30 mol-% [NEt_4][$C_5(CF_3)_4H$], 70 mol-% [NEt_4][$C_5(CF_3)_5$)]), corresponding to [NEt_4][$C_5(CF_3)_5$] (0.15 g, 0.28 mmol) was dissolved together with [Rh(COD)Cl]$_2$ (99 mg, 0.20 mmol, 0.50 equiv.) in anhydrous CH_2Cl_2 (2 mL). A suspension of $AgBF_4$ (80 mg, 0.41 mmol, 1.0 equiv.) in anhydrous CH_2Cl_2 (2 mL) was added dropwise at −20 °C and stirred at this temperature for 15 min, giving a yellow suspension. The solvent was removed in high vacuum and the residue was extracted with perfluorohexanes (3 × 1 mL) and filtrated under an argon atmosphere. Then the solvent was removed in high vacuum and the remaining solid was dissolved in anhydrous *n*-pentane (3 × 0.5 mL) and precipated at −100 °C. The residue was decanted and the solvent was removed in high vacuum to give the product [Rh(COD)($C_5(CF_3)_5$)] (0.10 g, 0.16 mmol) as a yellow solid. Referring to the applied amount of substance of [NEt_4][$C_5(CF_3)_5$], a yield of 58% is obtained.

^{1}H-NMR (401 MHz, $CDCl_3$, rt) δ [ppm] = 4.68 (s, 4H), 2.40–2.38 (m, 4H), 2.13–2.17 (m, 4H). **^{13}C{^{1}H}-NMR** (151 MHz, $CDCl_3$, rt) δ [ppm] = 77.7 (d, $^1J_{C,Rh}$ = 13.3 Hz, 4C), 31.5 (s, 4C). **^{13}C{^{19}F}-NMR** (151 MHz, $CDCl_3$, rt) δ [ppm] = 121.0 (s, 5C), 96.5 (d, $^1J_{C,Rh}$ = 3.4 Hz, 5C). **^{19}F-NMR** (377 MHz, $CDCl_3$, rt) δ [ppm] = –51.1 (s, 15F). **FT-IR** (ATR) $\tilde{\nu}$ [cm^{-1}] = 637 (vs), 697 (w), 750 (w), 784 (w), 809 (w), 824 (m), 882 (m), 946 (w), 979 (m), 1006 (m), 1154 (vs), 1190 (vs), 1426 (m), 2845 (w), 2895 (w), 2928 (w), 2956 (w). **HRMS** (EI-TOF, positive) m/z for $[C_{18}H_{12}F_{15}Rh]^+$ calculated: 615.9755; measured: 615.9758. **EA** ($C_{18}H_{12}F_{15}Rh$) calculated: C: 35.09%, H: 1.96%; measured: C: 35.28%, H: 2.33%.

Reversible formation of η^2,η^2-(Cycloocta-1,5-diene)-η^6-1,2,3,4,5,6-toluenerhodium(I) 1,2,3,4,5-pentakis(trifluoromethyl)cyclopentadienide from η^2,η^2-(Cycloocta-1,5-diene)-η^5-1,2,3,4,5-pentakis(trifluoromethyl)cyclopentadienylrhodium(I)

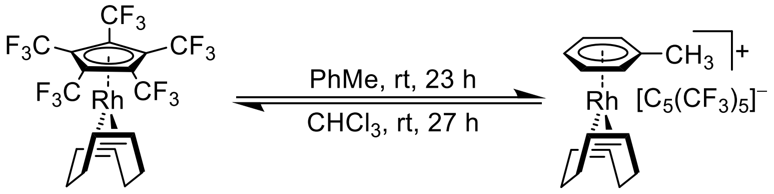

In a dried 10 mL Schlenk flask $[Rh(COD)(C_5(CF_3)_5]$ (40 mg, 65 µmol, 1.0 equiv.) was dissolved in anhydrous PhMe (2 mL) and stirred for 23 h at rt. Then, the solvent was removed in high vacuum and the residue was washed with anhydrous *n*-pentane (3 × 1 mL). The solvent was again removed in high vacuum to give the product $[Rh(COD)(PhMe)][C_5(CF_3)_5]$ (46 mg, 65 µmol) as a pale yellow solid in quantitative yield. The reaction progress was monitored over time by ^{19}F-NMR spectroscopy in d_8-PhMe (see NMR spectra).

$[Rh(COD)(PhMe)][C_5(CF_3)_5]$ (40 mg, 57 µmol, 1.0 equiv.) was dissolved in anhydrous $CHCl_3$ (2 mL) and stirred for 27 h at rt. Then, the solvent was removed in high vacuum to give the product $[Rh(COD)(C_5(CF_3)_5]$ (35 mg, 57 µmol) as a yellow solid in quantitative yield. The reaction progress was monitored over time by ^{1}H-NMR and ^{19}F-NMR spectroscopy in $CDCl_3$ (see NMR spectra).

[Rh(COD)(PhMe)][$C_5(CF_3)_5$]: **^{1}H-NMR** (401 MHz, CD_2Cl_2, rt) δ [ppm] = 6.57–6.47 (m, 5H), 4.51 (s, 4H), 2.34–2.30 (m, 7H), 2.13–2.11 (m, 4H). **^{13}C{^{1}H}-NMR** (151 MHz, CD_2Cl_2, rt) δ [ppm] = 121.1 (d, $^1J_{C,Rh}$ = 2.1 Hz, 1C), 106.1 (d, $^1J_{C,Rh}$ = 2.7 Hz, 2C), 104.7 (d, $^1J_{C,Rh}$ = 2.7 Hz, 2C), 103.1 (d, $^1J_{C,Rh}$ = 2.6 Hz, 1C), 79.8 (d, $^1J_{C,Rh}$ = 12.5 Hz, 4C), 31.6 (s, 4C), 19.5 (s, 1C). **^{13}C{^{19}F}-NMR** (151 MHz, CD_2Cl_2, rt) δ [ppm] = 121.3 (s, 5C), 110.2 (s, 5C). **^{19}F-NMR** (377 MHz, CD_2Cl_2, rt) δ [ppm] = –50.6 (s, 15F). **FT-IR** (ATR) $\tilde{\nu}$ [cm^{-1}] = 613 (s), 695 (w), 802 (m), 826 (w), 884 (w), 985 (m), 1000 (m), 1007 (vs), 1200 (vs), 1437 (w), 1493 (m), 2851 (w), 2894 (w), 2931 (w), 2968 (w), 3011 (w). **HRMS** (ESI-TOF, positive) m/z for $[C_7H_8Rh]^+$ calculated: 194.9681; measured: 194.9551. **HRMS** (ESI-TOF, negative) m/z for $[C_{10}F_{15}]^-$ calculated: 404.9766; measured: 404.9486. **EA** ($C_{25}H_{20}F_{15}Rh$) calculated: C: 42.39%, H: 2.85%; measured: C: 42.47%, H: 2.87%.

NMR Spectra

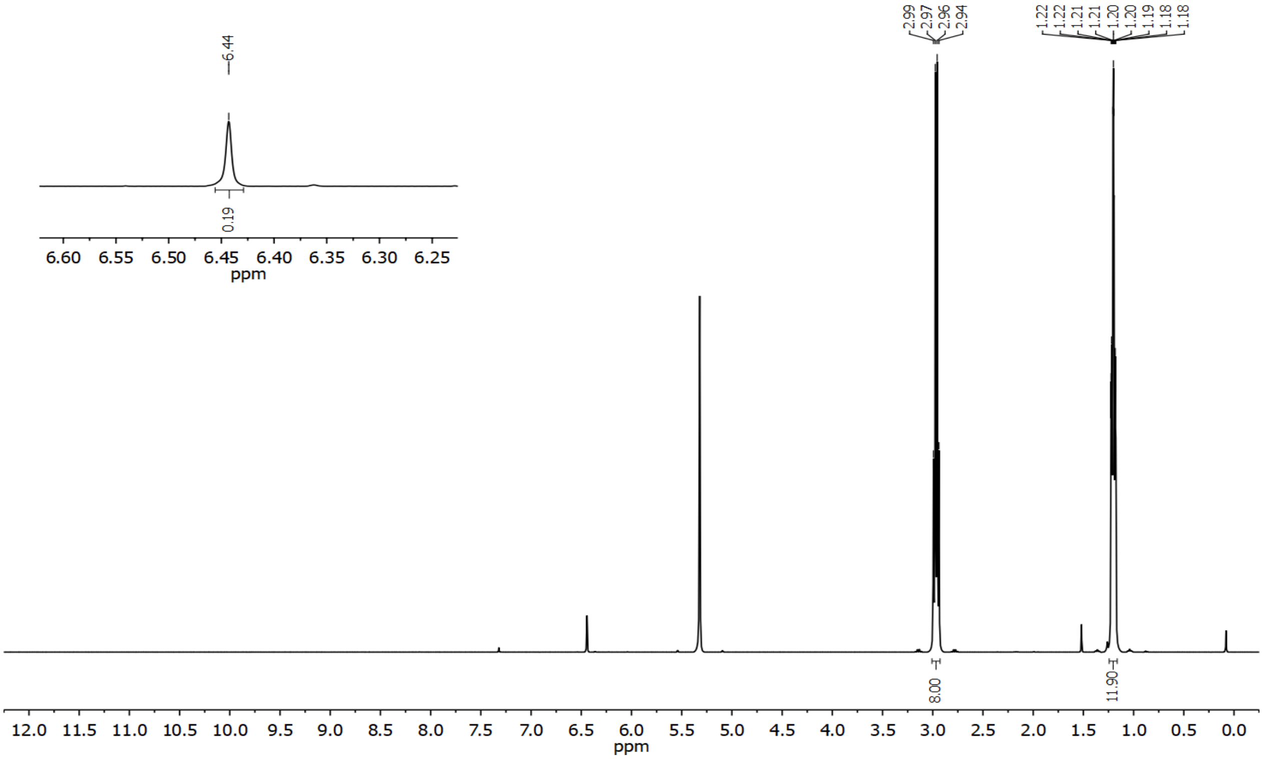

Figure 1. ^{1}H-NMR (401 MHz, CD_2Cl_2, rt) spectrum of $[NEt_4][C_5(CF_3)_5]$ with quantities of $[NEt_4][C_5(CF_3)_4H]$ (top-left).

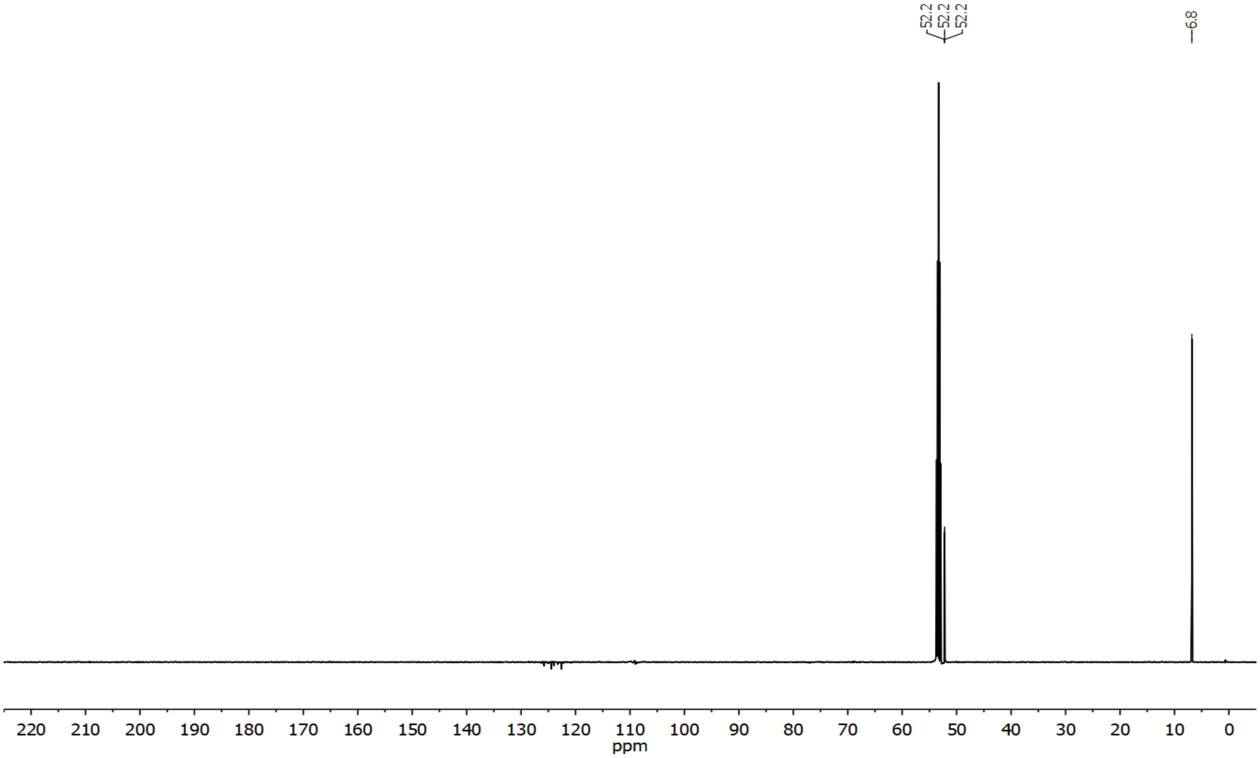

Figure 2. $^{13}C\{^{1}H\}$-NMR (151 MHz CD_2Cl_2, rt) spectrum of $[NEt_4][C_5(CF_3)_5]$.

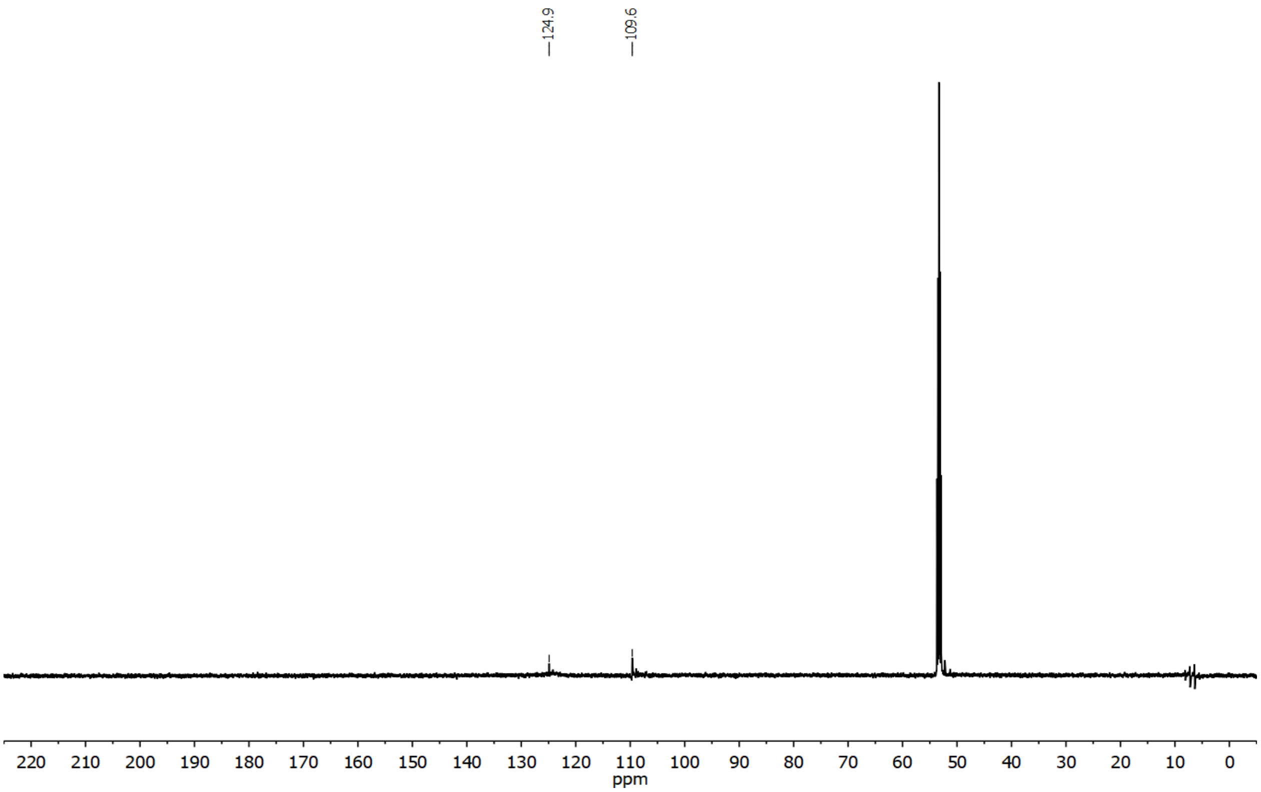

Figure 3. $^{13}C\{^{19}F\}$-NMR (151 MHz CD_2Cl_2, rt) spectrum of $[NEt_4][C_5(CF_3)_5]$.

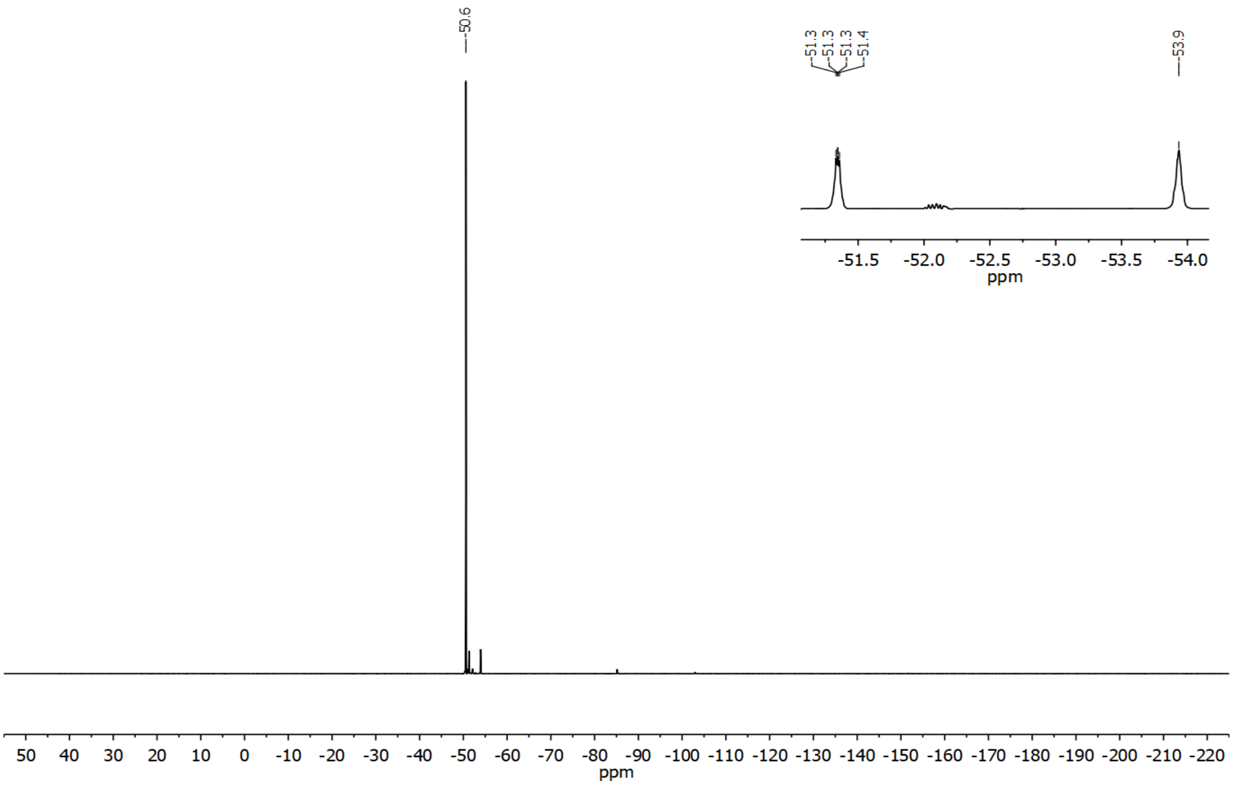

Figure 4. ^{19}F-NMR (377 MHz, CD_2Cl_2, rt) spectrum of $[NEt_4][C_5(CF_3)_5]$ with quantities of $[NEt_4][C_5(CF_3)_4H]$ (top-right).

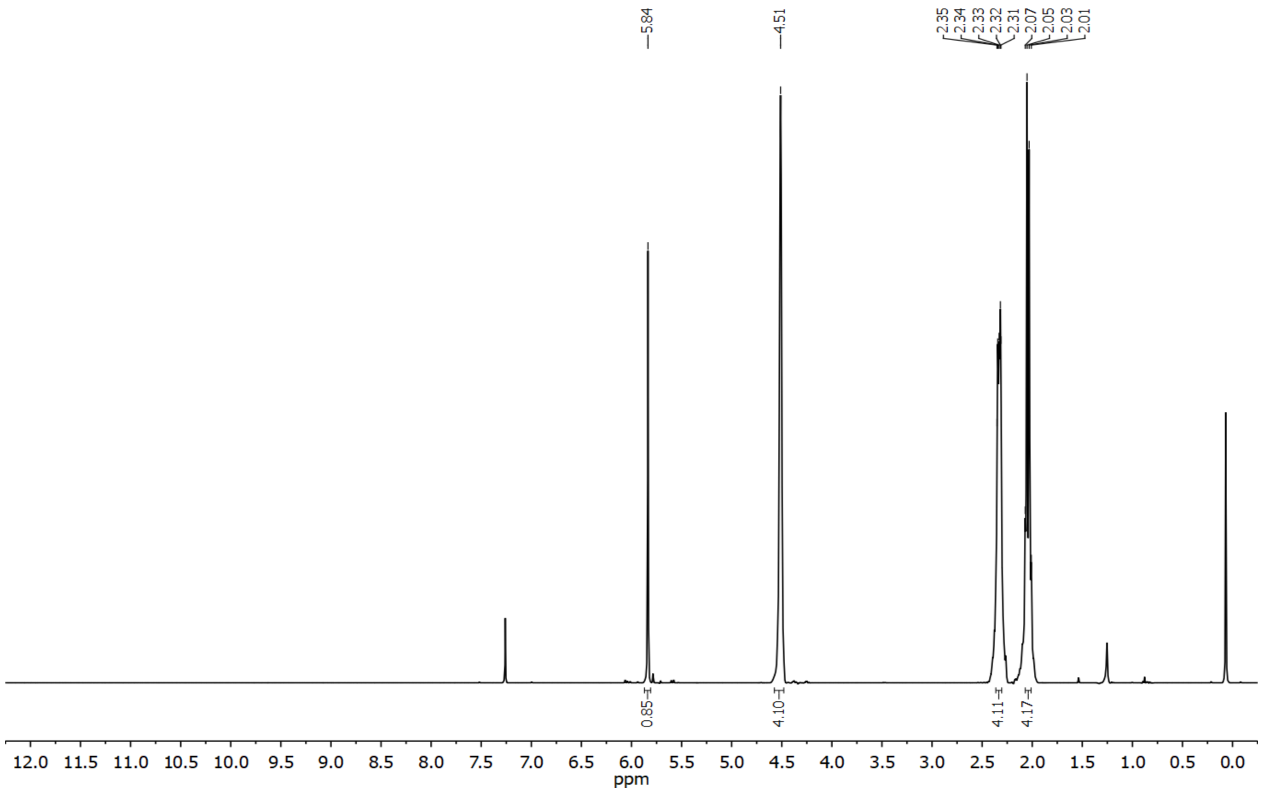

Figure 5. ^{1}H-NMR (401 MHz, $CDCl_3$, rt) spectrum of [Rh(COD)($C_5(CF_3)_4H$)].

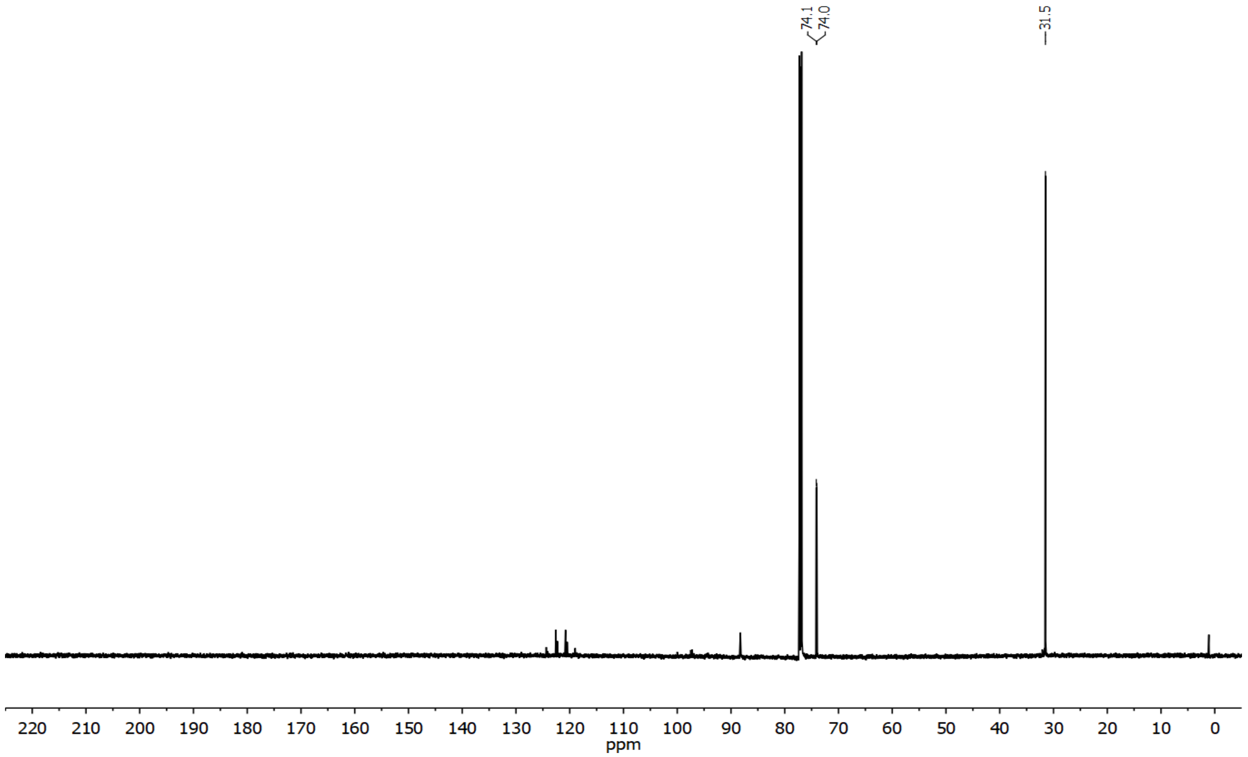

Figure 6. $^{13}C\{^{1}H\}$-NMR (151 MHz, $CDCl_3$, rt) spectrum of [Rh(COD)($C_5(CF_3)_4H$)].

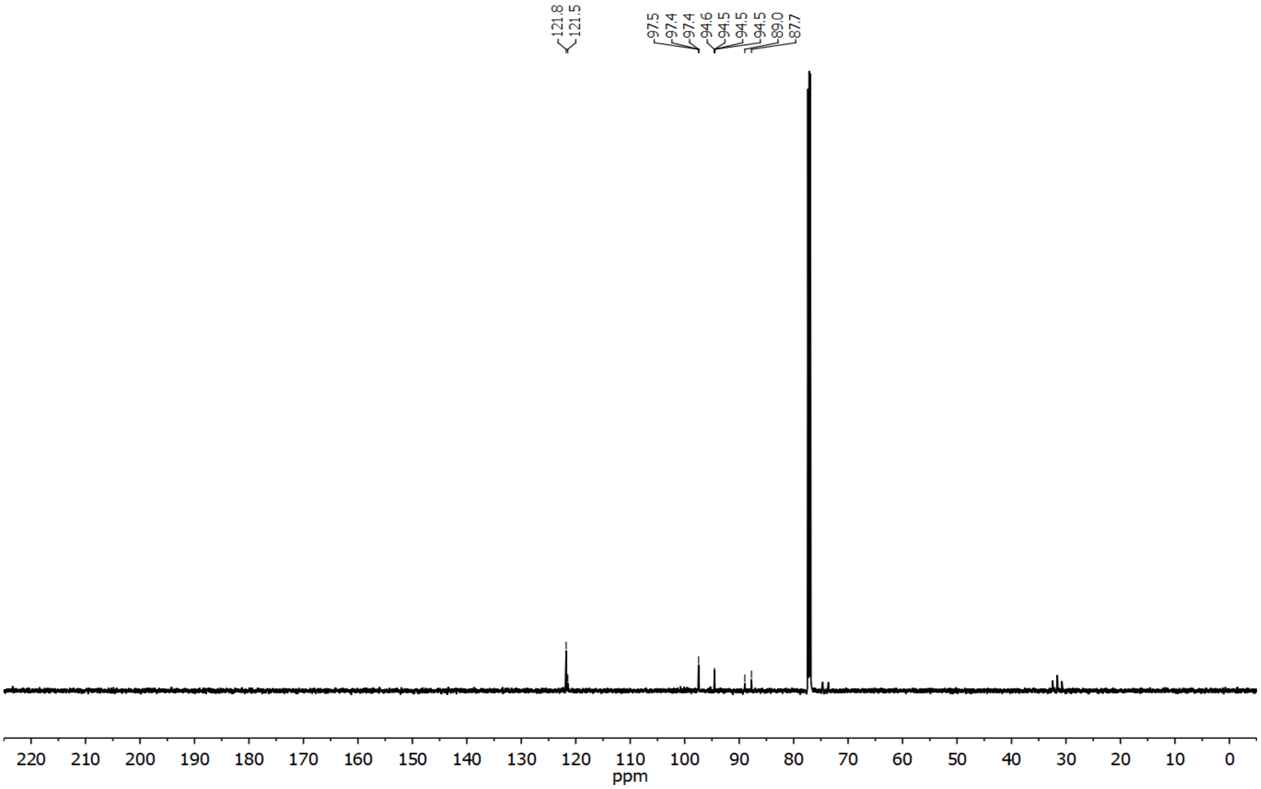

Figure 7. $^{13}C\{^{19}F\}$-NMR (151 MHz, $CDCl_3$, rt) spectrum of [Rh(COD)($C_5(CF_3)_4H$)].

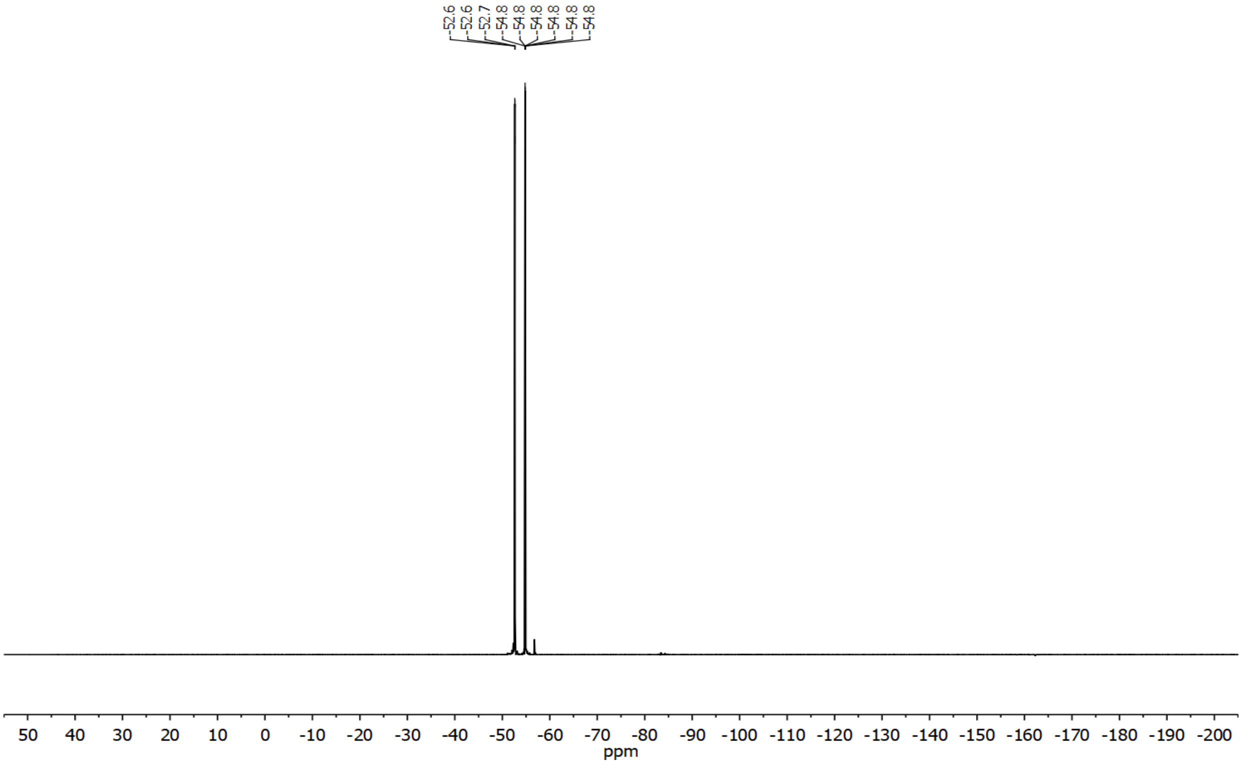

Figure 8. ^{19}F-NMR (377 MHz, $CDCl_3$, rt) spectrum of [Rh(COD)($C_5(CF_3)_4H$)].

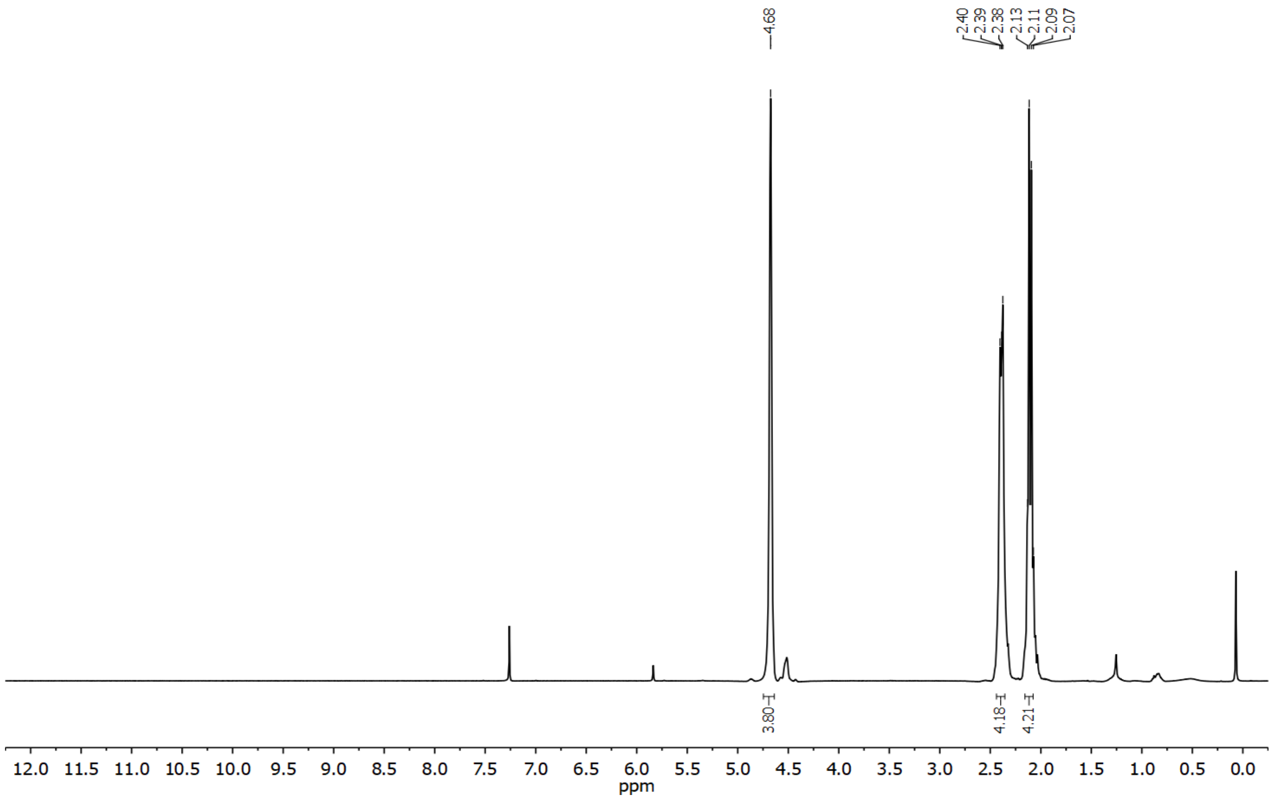

Figure 9. ^{1}H-NMR (401 MHz, $CDCl_3$, rt) spectrum of [Rh(COD)($C_5(CF_3)_5$)].

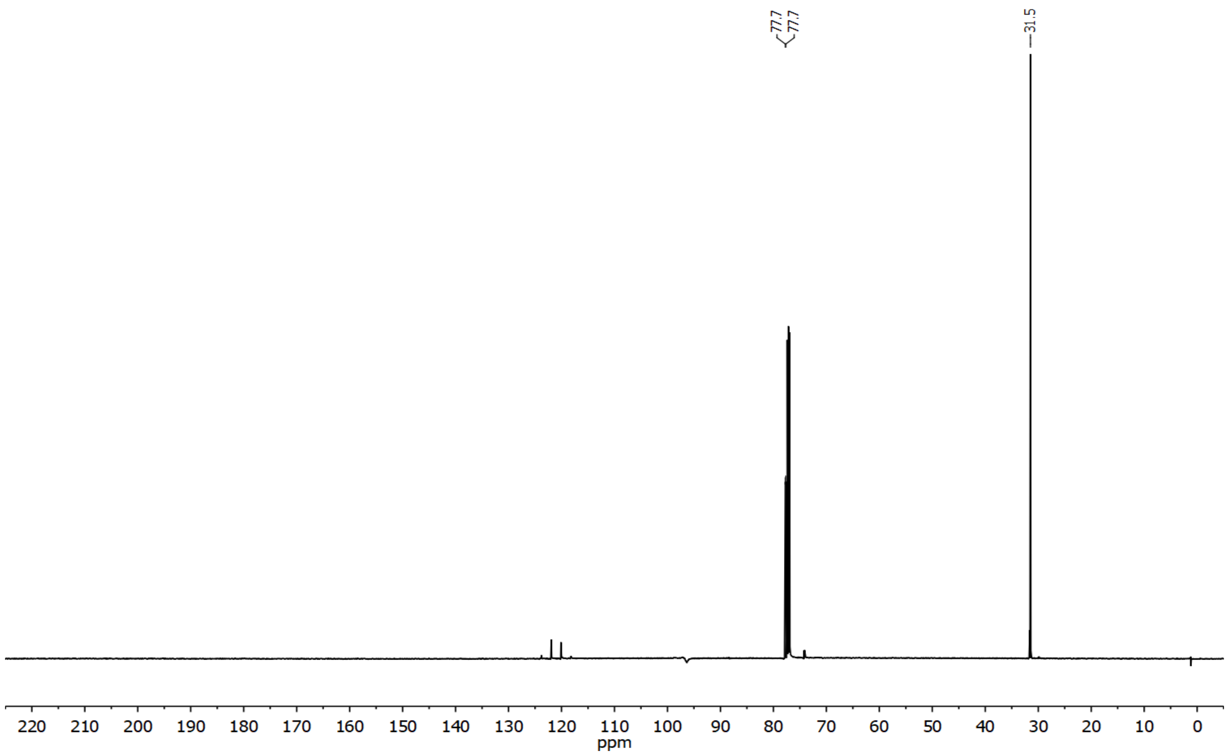

Figure 10. ^{13}C{^{1}H}-NMR (151 MHz, $CDCl_3$, rt) spectrum of [Rh(COD)($C_5(CF_3)_5$)].

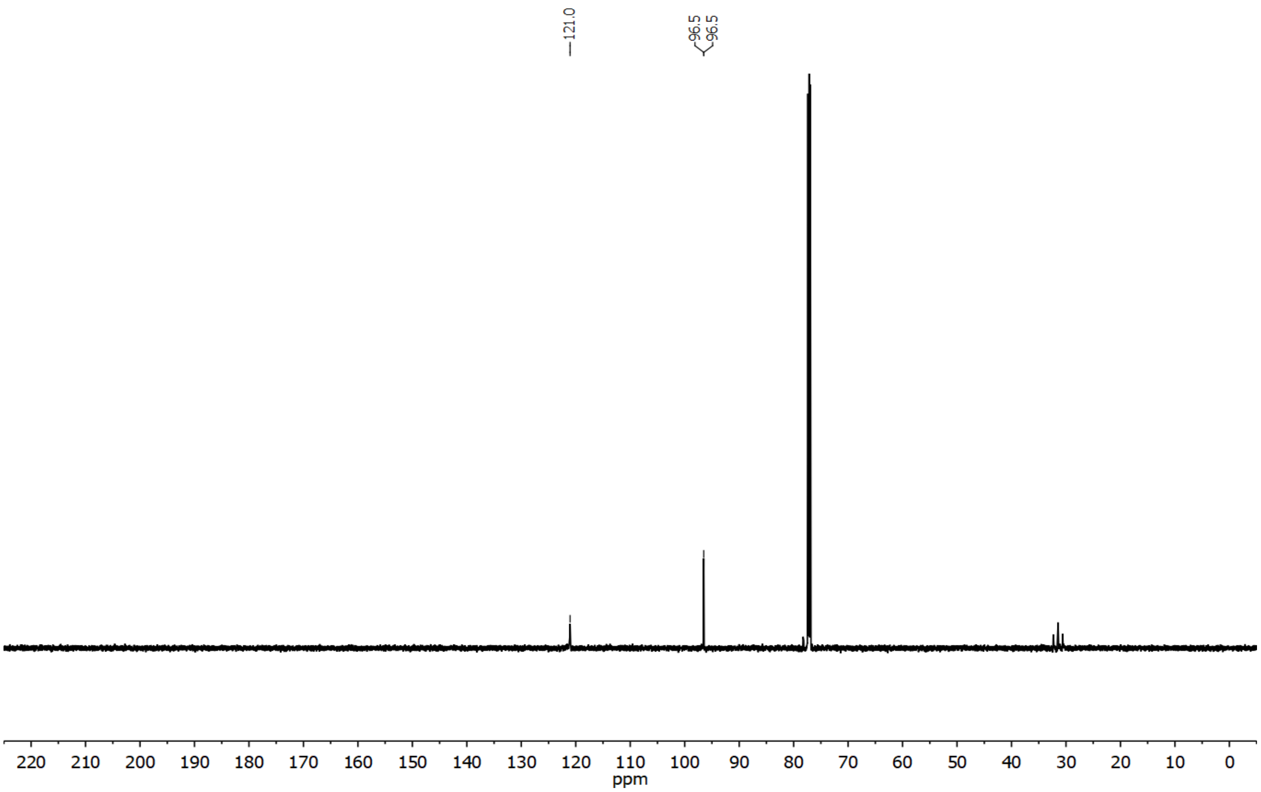

Figure 11. $^{13}C\{^{19}F\}$-NMR (151 MHz, $CDCl_3$, rt) spectrum of [Rh(COD)($C_5(CF_3)_5$)].

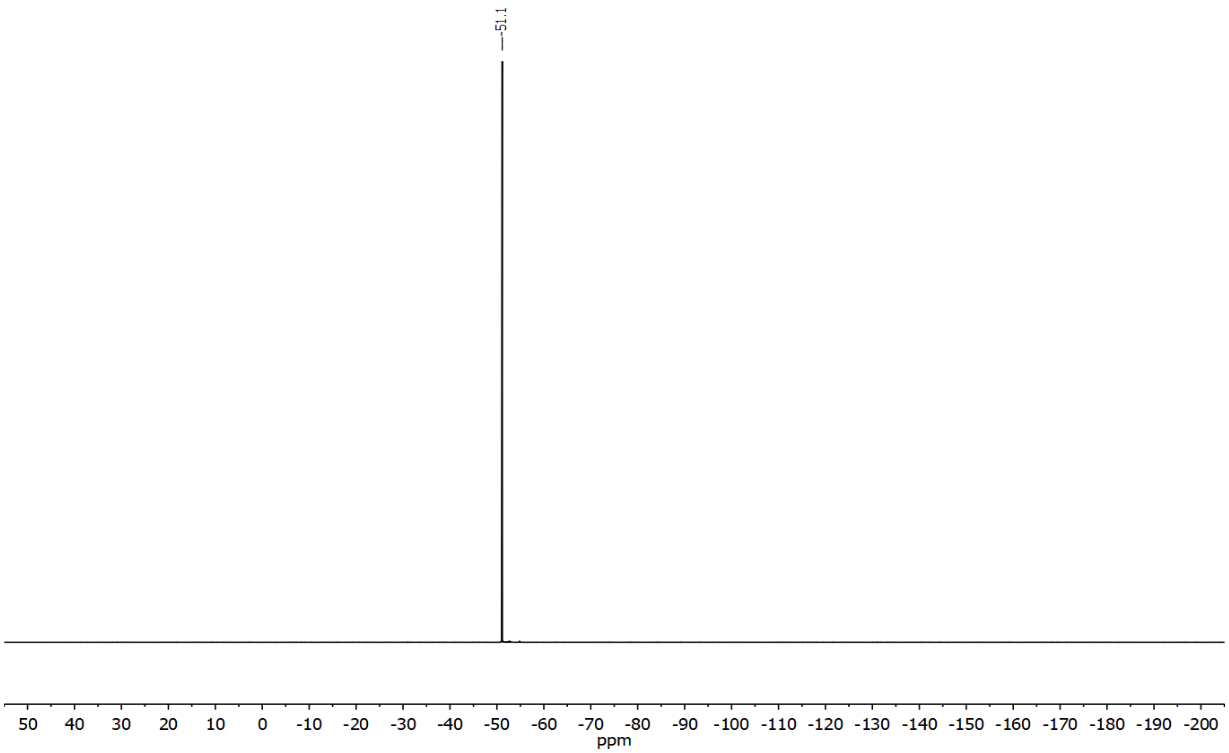

Figure 12. ^{19}F-NMR (377 MHz, $CDCl_3$, rt) spectrum of [Rh(COD)($C_5(CF_3)_5$)].

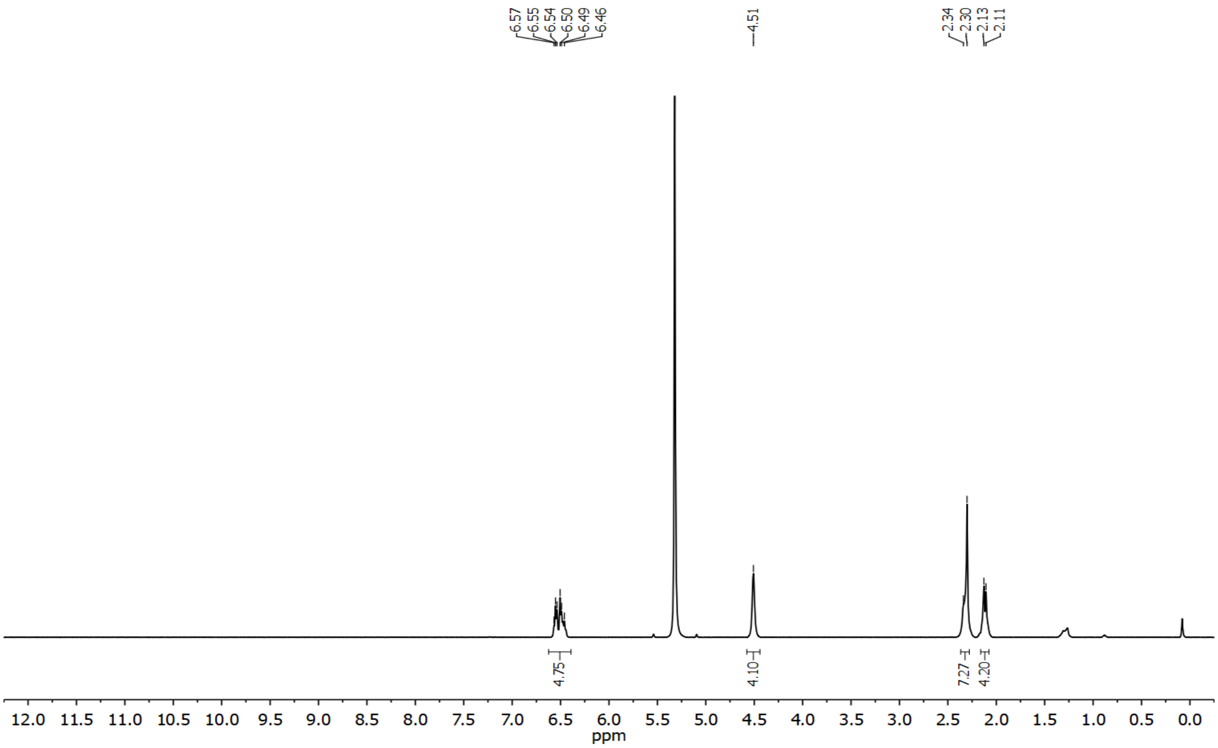

Figure 13. ^{1}H-NMR (401 MHz, CD_2Cl_2, rt) spectrum of [Rh(COD)(PhMe)][$C_5(CF_3)_5$].

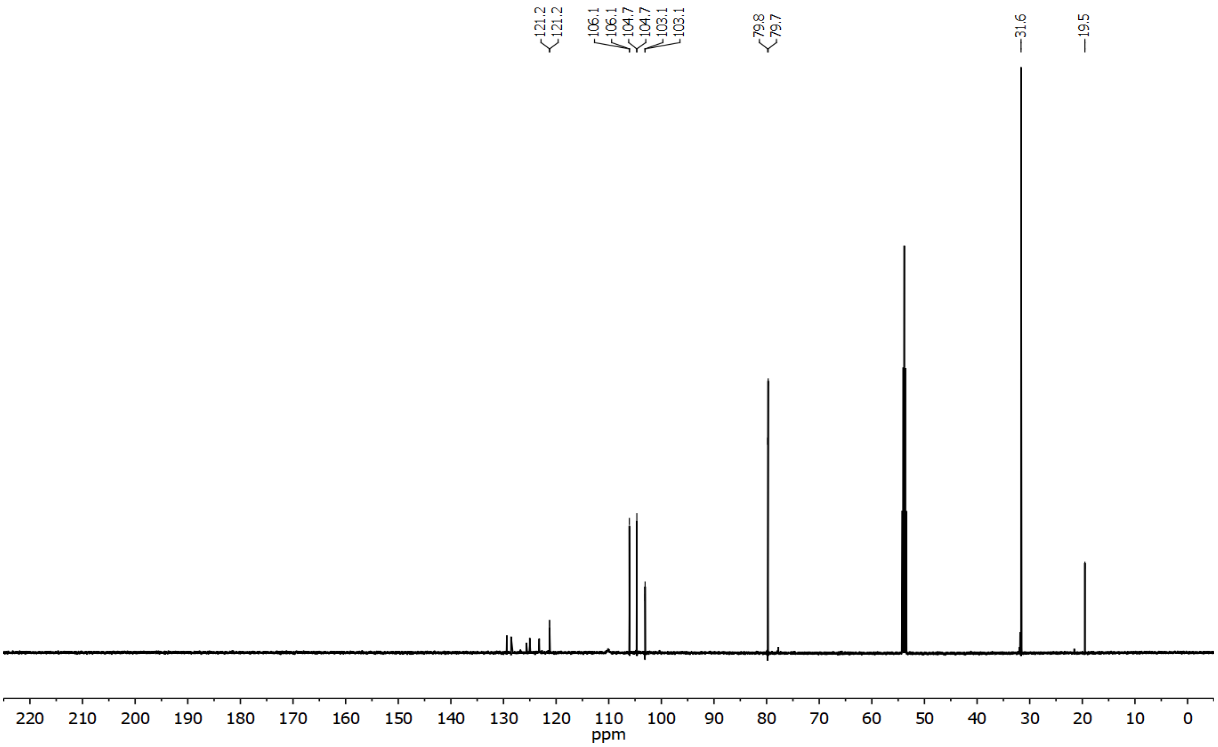

Figure 14. ^{13}C{^{1}H}-NMR (151 MHz, CD_2Cl_2, rt) spectrum of [Rh(COD)(PhMe)][$C_5(CF_3)_5$].

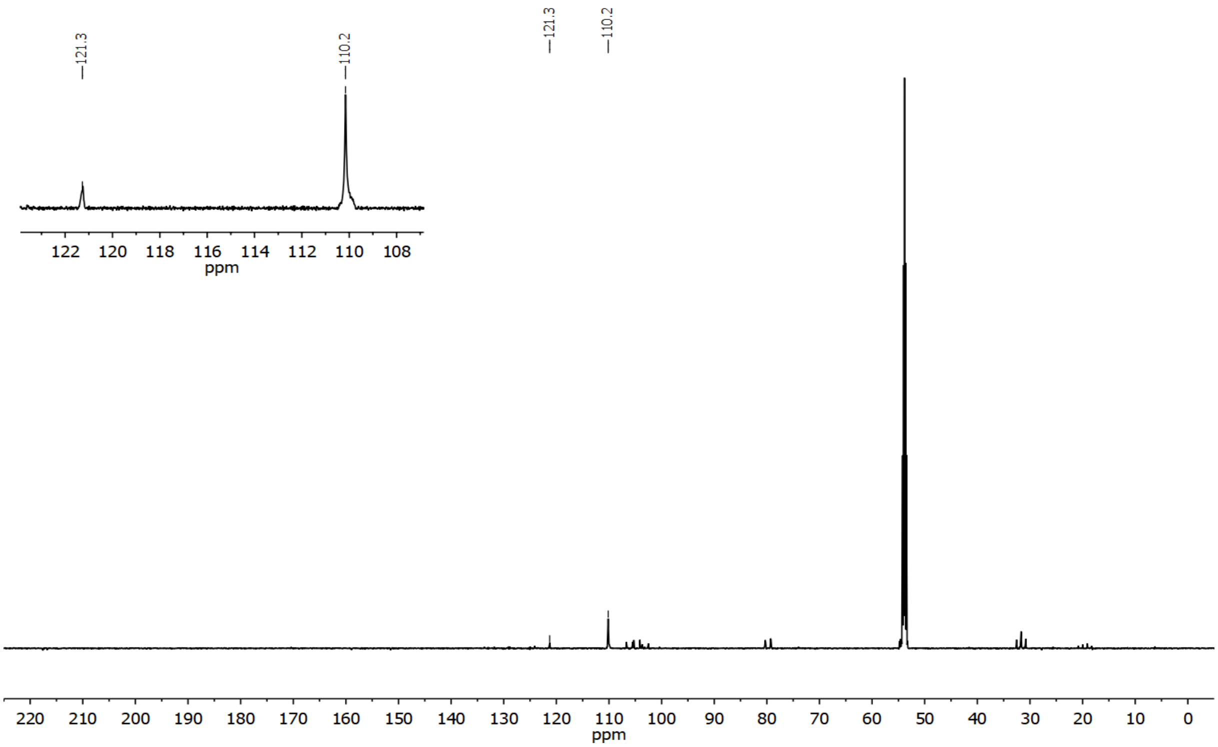

Figure 15. $^{13}C\{^{19}F\}$-NMR (151 MHz, CD_2Cl_2, rt) spectrum of $[Rh(COD)(PhMe)][C_5(CF_3)_5]$.

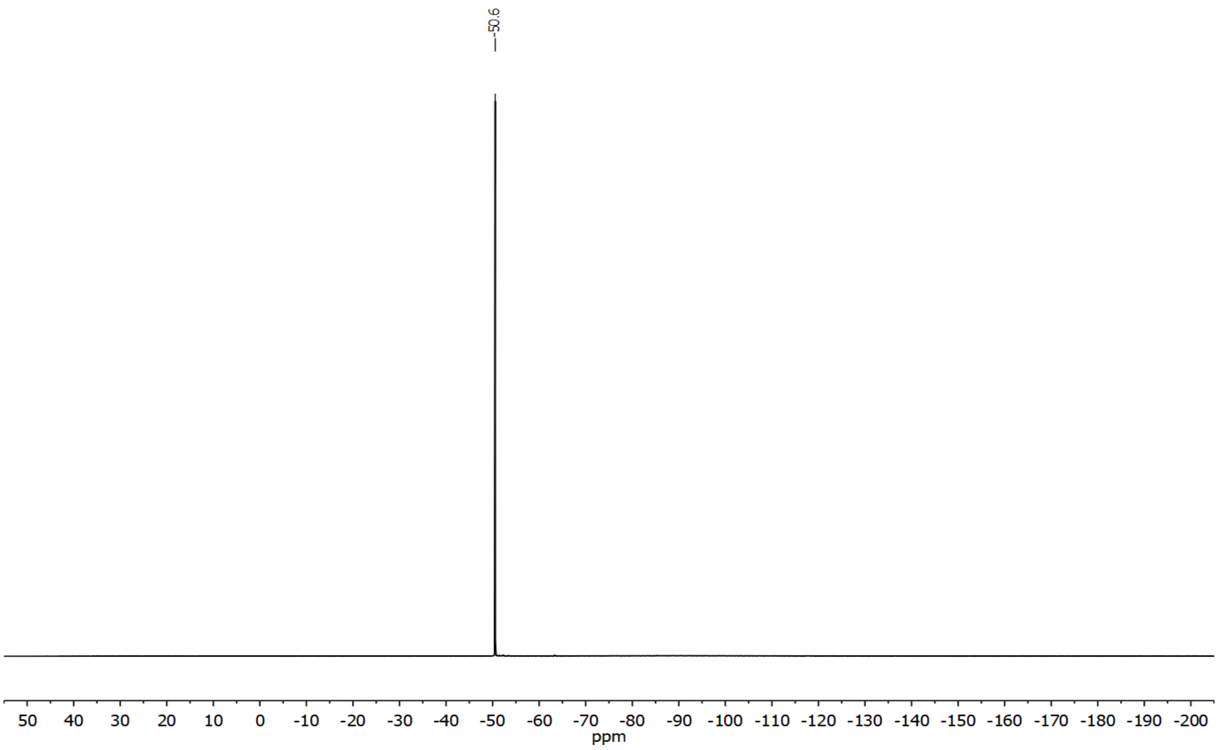

Figure 16. ^{19}F-NMR (377 MHz, CD_2Cl_2, rt) spectrum of $[Rh(COD)(PhMe)][C_5(CF_3)_5]$.

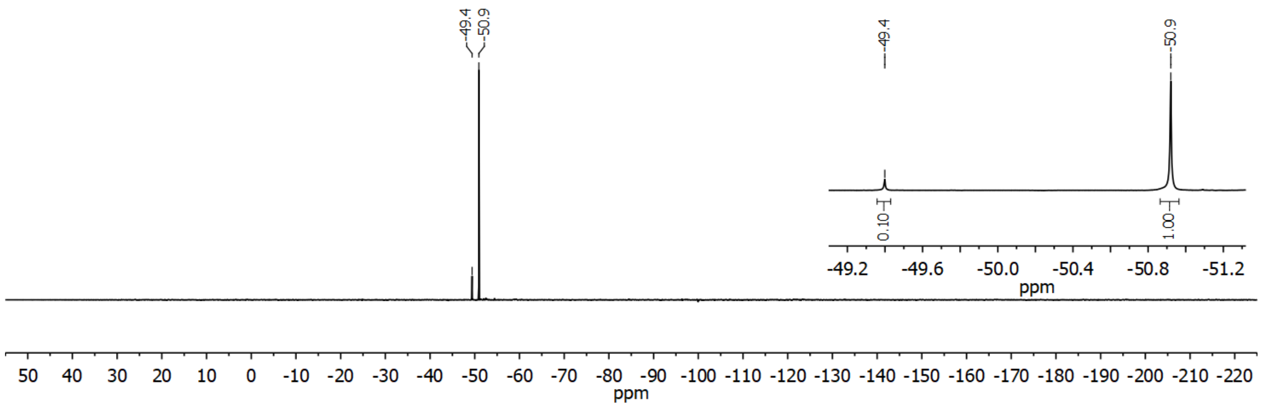

Figure 17. ^{19}F-NMR (377 MHz, d_8-PhMe, rt) reaction control of $[Rh(COD)(C_5(CF_3)_5)]$ to $[Rh(COD)(d_8\text{-}PhMe)][C_5(CF_3)_5]$ after 10 min.

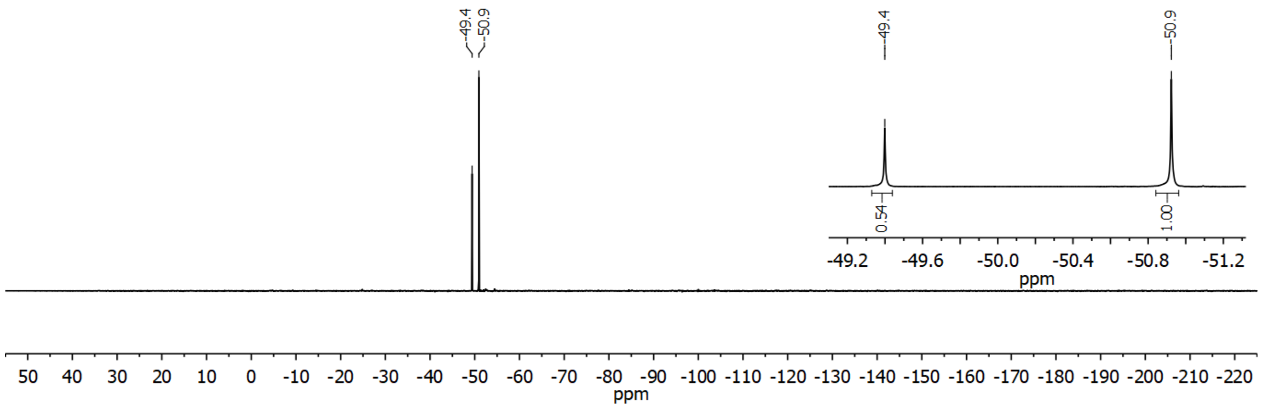

Figure 18. ^{19}F-NMR (377 MHz, d_8-PhMe, rt) reaction control of $[Rh(COD)(C_5(CF_3)_5)]$ to $[Rh(COD)(d_8\text{-}PhMe)][C_5(CF_3)_5]$ after 1 h.

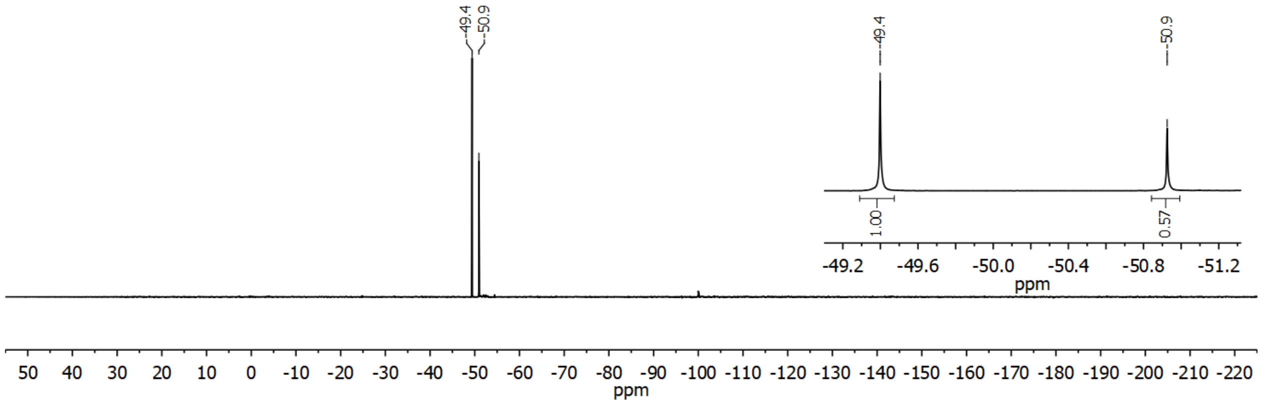

Figure 19. ^{19}F-NMR (377 MHz, d_8-PhMe, rt) reaction control of $[Rh(COD)(C_5(CF_3)_5)]$ to $[Rh(COD)(d_8\text{-}PhMe)][C_5(CF_3)_5]$ after 3 h.

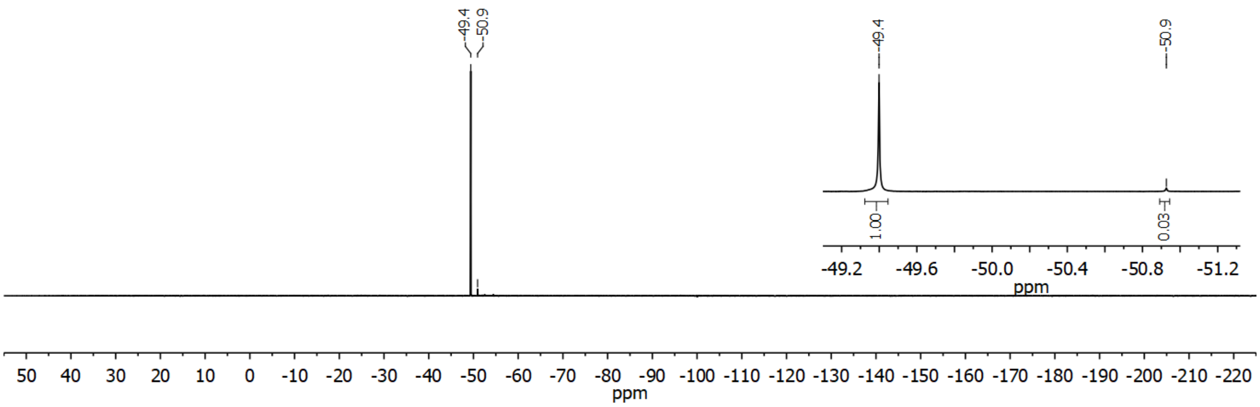

Figure 20. ^{19}F-NMR (377 MHz, d_8-PhMe, rt) reaction control of [Rh(COD)($C_5(CF_3)_5$)] to [Rh(COD)(d_8-PhMe)][$C_5(CF_3)_5$] after 23 h.

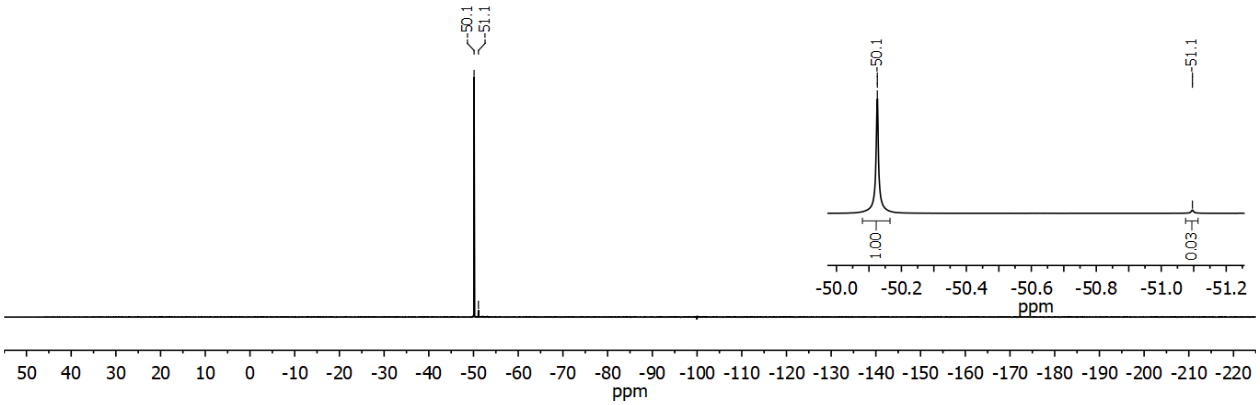

Figure 21. ^{19}F-NMR (377 MHz, $CDCl_3$, rt) reaction control of [Rh(COD)(PhMe)][$C_5(CF_3)_5$] to [Rh(COD)($C_5(CF_3)_5$)] after 10 min.

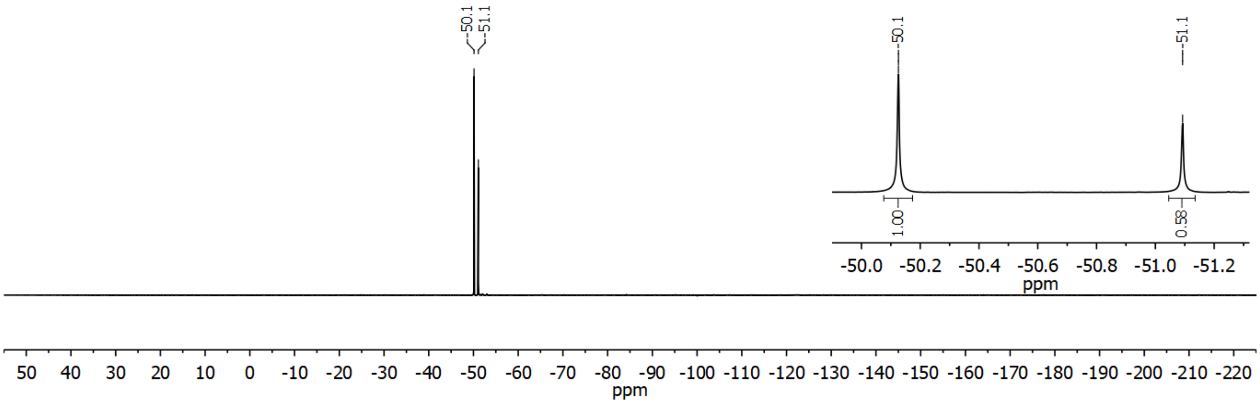

Figure 22. ^{19}F-NMR (377 MHz, $CDCl_3$, rt) reaction control of [Rh(COD)(PhMe)][$C_5(CF_3)_5$] to [Rh(COD)($C_5(CF_3)_5$)] after 6 h.

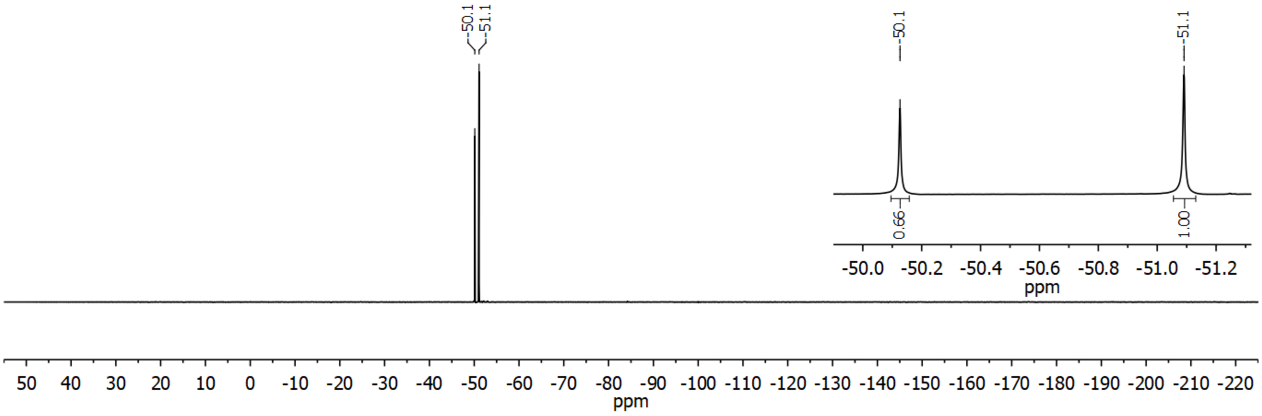

Figure 23. ^{19}F-NMR (377 MHz, $CDCl_3$, rt) reaction control of [Rh(COD)(PhMe)][$C_5(CF_3)_5$] to [Rh(COD)($C_5(CF_3)_5$)] after 12 h.

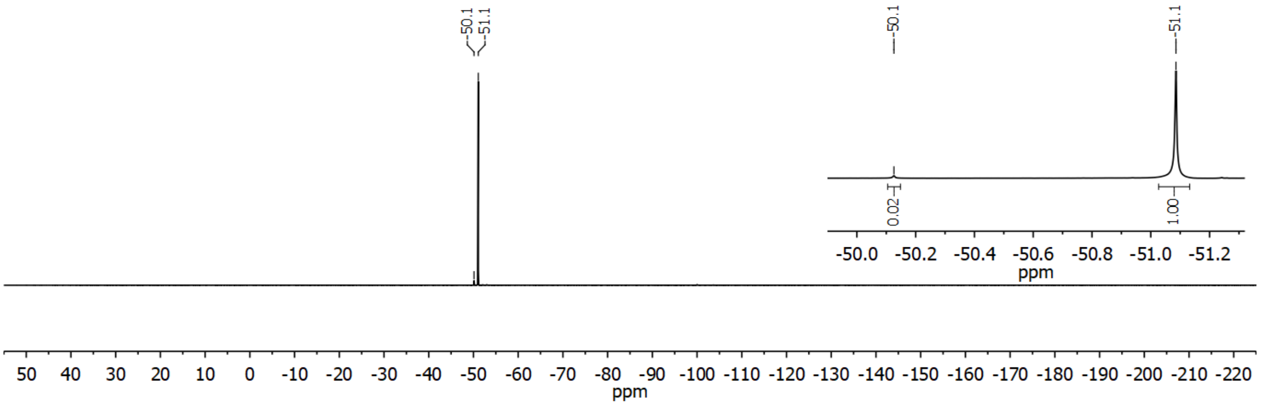

Figure 24. ^{19}F-NMR (377 MHz, $CDCl_3$, rt) reaction control of [Rh(COD)(PhMe)][$C_5(CF_3)_5$] to [Rh(COD)($C_5(CF_3)_5$)] after 27h.

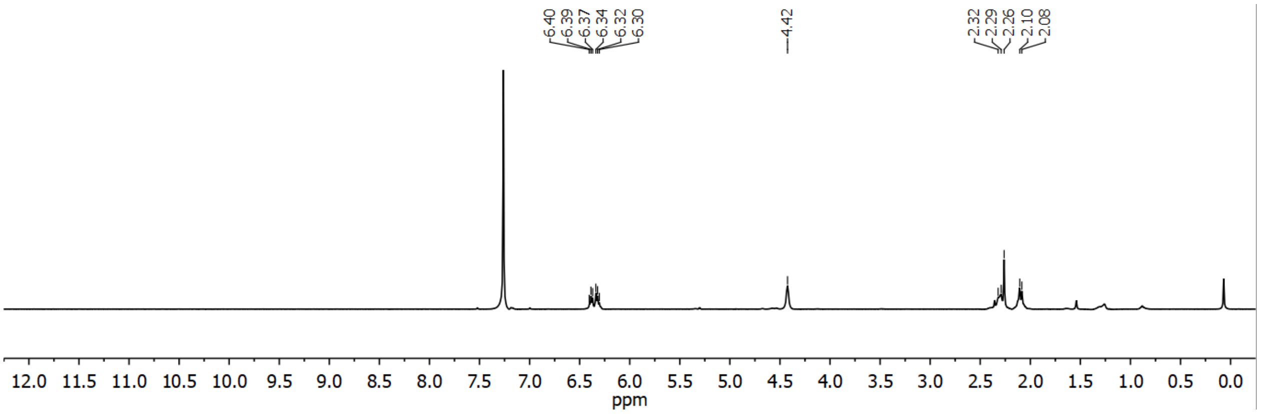

Figure 25. ^{1}H-NMR (401 MHz, $CDCl_3$, rt) reaction control of [Rh(COD)(PhMe)][$C_5(CF_3)_5$] to [Rh(COD)($C_5(CF_3)_5$)] after 10 min.

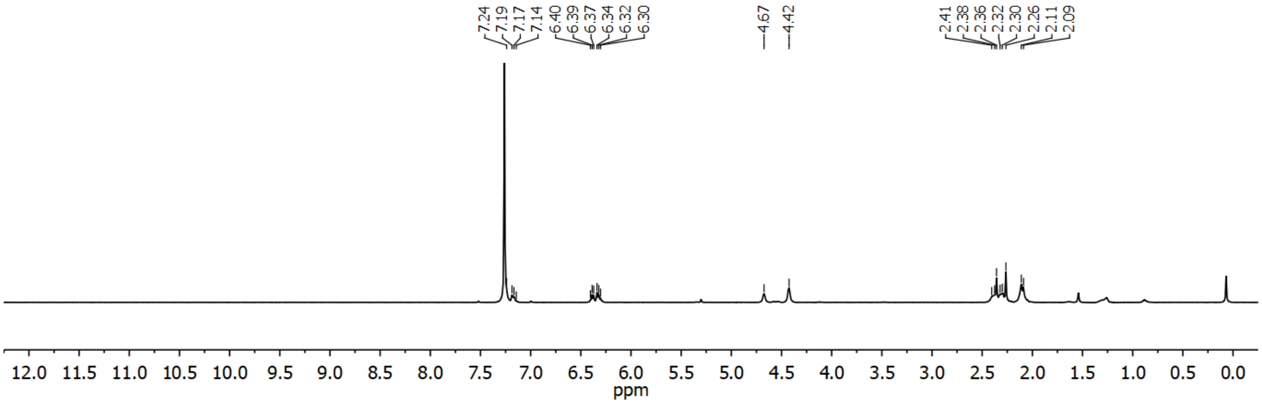

Figure 26. ^{1}H-NMR (401 MHz, $CDCl_3$, rt) reaction control of [Rh(COD)(PhMe)][$C_5(CF_3)_5$] to [Rh(COD)($C_5(CF_3)_5$)] after 6 h.

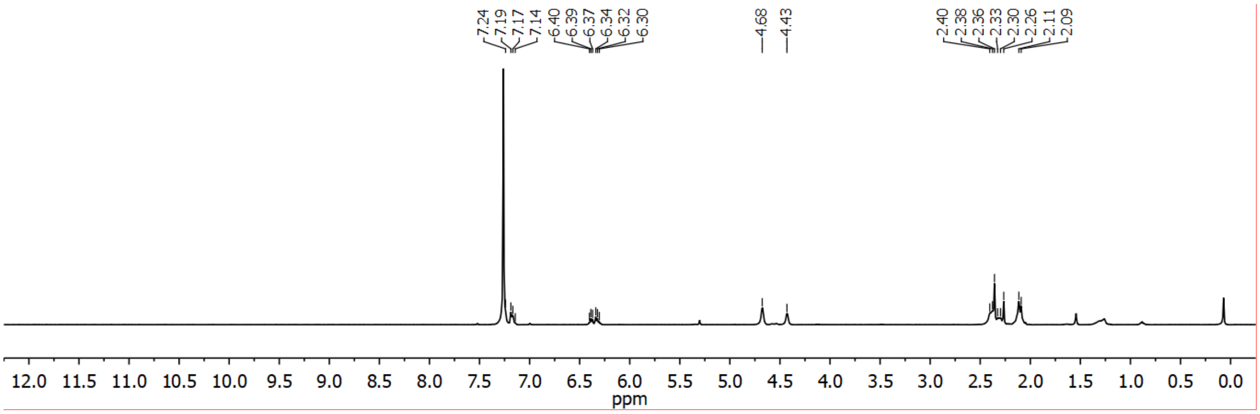

Figure 27. ^{1}H-NMR (401 MHz, $CDCl_3$, rt) reaction control of [Rh(COD)(PhMe)][$C_5(CF_3)_5$] to [Rh(COD)($C_5(CF_3)_5$)] after 12 h.

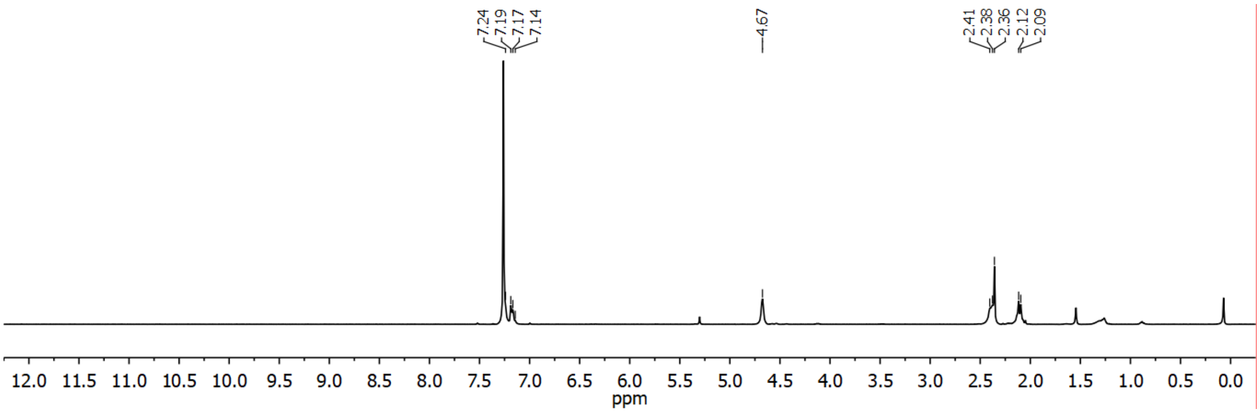

Figure 28. ^{1}H-NMR (401 MHz, $CDCl_3$, rt) reaction control of [Rh(COD)(PhMe)][$C_5(CF_3)_5$] to [Rh(COD)($C_5(CF_3)_5$)] after 27 h.

IR Spectra

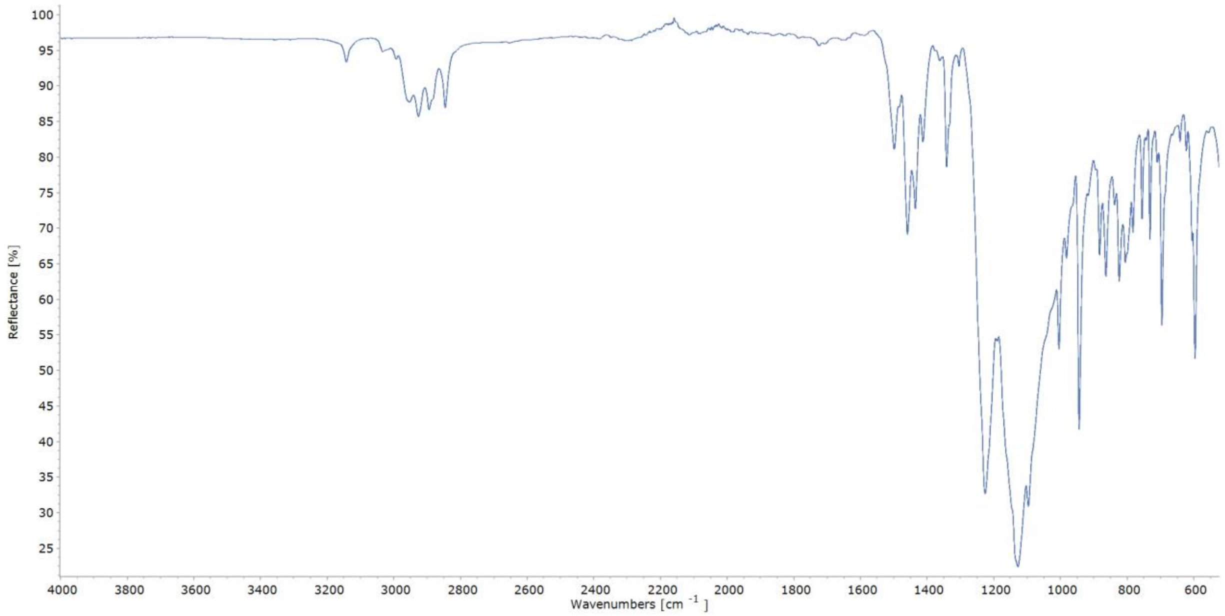

Figure 29. IR (ATR, rt) spectrum of [Rh(COD)($C_5(CF_3)_4H$)].

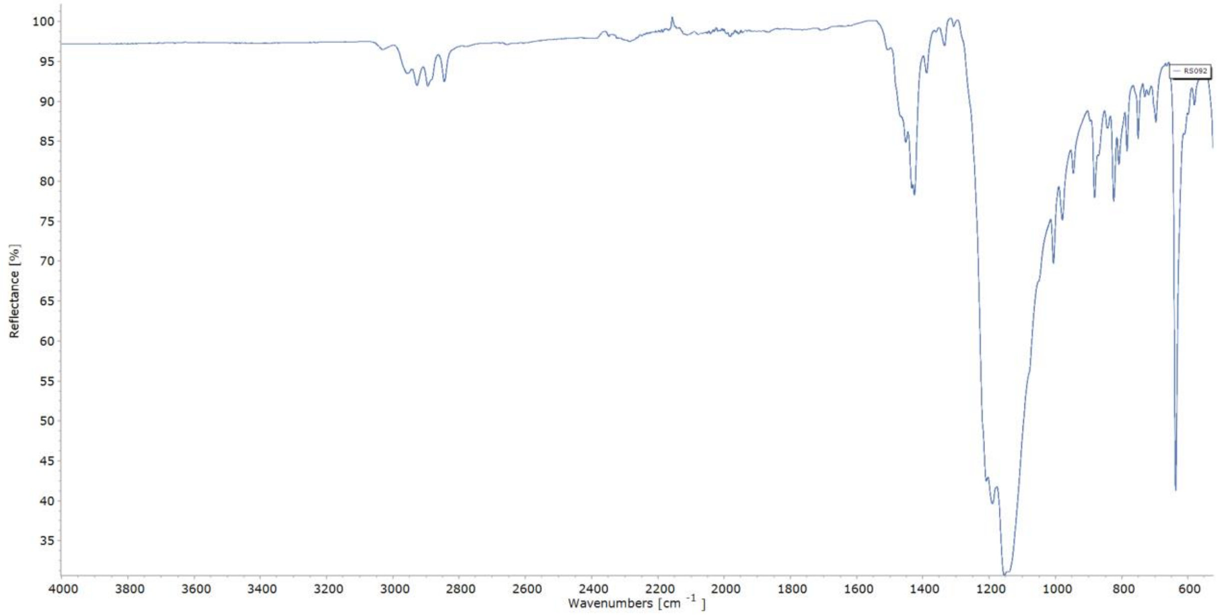

Figure 30. IR (ATR, rt) spectra of [Rh(COD)($C_5(CF_3)_5$)].

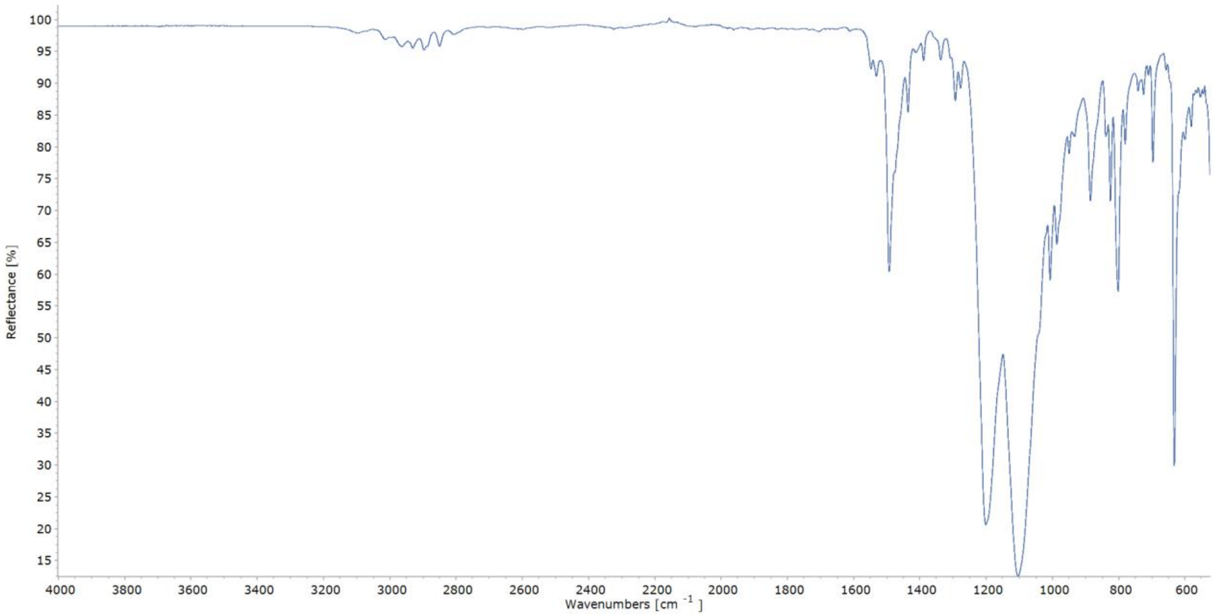

Figure 31. IR (ATR, rt) spectra of $[Rh(COD)(PhMe)][C_5(CF_3)_5]$.

Crystallographic Data

Table 1. Crystallographic data of [Rh(COD)($C_5(CF_3)_4H$)].

Identification code	CCDC 2169582
Empirical formula	$C_{17}H_{13}F_{12}Rh$
Formula weight	548.18
Temperature/K	100
Crystal system	monoclinic
Space group	$P2_1/n$
a/Å	9.038(3)
b/Å	13.516(3)
c/Å	14.292(3)
α/°	90
β/°	93.008(11)
γ/°	90
Volume/$Å^3$	1743.4(7)
Z	4
$\rho_{calc}g/cm^3$	2.088
μ/mm^{-1}	1.104
F(000)	1072.0
Crystal size/mm^3	0.29 × 0.25 × 0.23
Radiation	MoKα (λ = 0.71073)
2Θ range for data collection/°	4.15 to 57.172
Index ranges	-12 ≤ h ≤ 12, -18 ≤ k ≤ 18, -19 ≤ l ≤ 19
Reflections collected	28148
Independent reflections	4417 [R_{int} = 0.0722, R_{sigma} = 0.0490]
Data/restraints/parameters	4417/0/271
Goodness-of-fit on F^2	1.060
Final R indexes [I>=2σ (I)]	R_1 = 0.0420, wR_2 = 0.1053
Final R indexes [all data]	R_1 = 0.0528, wR_2 = 0.1123
Largest diff. peak/hole / e $Å^{-3}$	1.43/-1.36

Table 2. Crystallographic data of [Rh(COD)($C_5(CF_3)_5$)] · 0.25 *n*-pentane.

Identification code	CCDC 2169581
Empirical formula	$C_{77}H_{58}F_{60}Rh_4$
Formula weight	2534.87
Temperature/K	100.0
Crystal system	triclinic
Space group	P-1
a/Å	13.4973(17)
b/Å	13.757(2)
c/Å	13.943(2)
α/°	62.112(6)
β/°	64.122(4)
γ/°	78.626(6)
Volume/Å^3	2058.8(5)
Z	1
ρ_{calc}g/cm^3	2.045
μ/mm^{-1}	0.970
F(000)	1240.0
Crystal size/mm^3	0.6 × 0.53 × 0.49
Radiation	MoKα (λ = 0.71073)
2Θ range for data collection/°	4.72 to 56.664
Index ranges	-17 ≤ h ≤ 18, -18 ≤ k ≤ 18, -18 ≤ l ≤ 18
Reflections collected	116644
Independent reflections	10211 [R_{int} = 0.0323, R_{sigma} = 0.0147]
Data/restraints/parameters	10211/4/921
Goodness-of-fit on F^2	1.076
Final R indexes [I>=2σ (I)]	R_1 = 0.0482, wR_2 = 0.1192
Final R indexes [all data]	R_1 = 0.0529, wR_2 = 0.1228
Largest diff. peak/hole / e Å^{-3}	1.61/-1.03

Table 3. Crystallographic data of [Rh(COD)($C_5(CF_3)_5$)].

Identification code	2193226
Empirical formula	$C_{36}H_{24}F_{30}Rh_2$
Formula weight	1232.37
Temperature/K	111.0
Crystal system	triclinic
Space group	P-1
a/Å	9.7219(8)
b/Å	13.4847(12)
c/Å	15.3398(14)
α/°	71.261(3)
β/°	89.466(3)
γ/°	88.527(3)
Volume/Å^3	1903.8(3)
Z	2
ρ_{calc}g/cm^3	2.150
μ/mm^{-1}	1.046
F(000)	1200.0
Crystal size/mm^3	0.6 × 0.38 × 0.22
Radiation	MoKα (λ = 0.71073)
2Θ range for data collection/°	4.19 to 52.83
Index ranges	-12 ≤ h ≤ 12, -15 ≤ k ≤ 16, 0 ≤ l ≤ 19
Reflections collected	7642
Independent reflections	7642 [R_{int} = ?, R_{sigma} = 0.0335]
Data/restraints/parameters	7642/0/614
Goodness-of-fit on F^2	1.079
Final R indexes [I>=2σ (I)]	R_1 = 0.0296, wR_2 = 0.0690
Final R indexes [all data]	R_1 = 0.0350, wR_2 = 0.0717
Largest diff. peak/hole / e Å^{-3}	0.71/-0.51

Table 4. Crystallographic data of [Rh(COD)(PhMe)][$C_5(CF_3)_5$].

Identification code	2193220
Empirical formula	$C_{25}H_{20}F_{15}Rh$
Formula weight	708.32
Temperature/K	102.0
Crystal system	monoclinic
Space group	Ia
a/Å	10.1030(4)
b/Å	14.1132(5)
c/Å	17.5257(8)
α/°	90
β/°	102.9900(10)
γ/°	90
Volume/Å^3	2434.96(17)
Z	4
ρ_{calc}g/cm^3	1.932
μ/mm^{-1}	0.832
F(000)	1400.0
Crystal size/mm^3	0.318 × 0.238 × 0.171
Radiation	MoKα (λ = 0.71073)
2Θ range for data collection/°	4.77 to 52.764
Index ranges	-12 ≤ h ≤ 12, -17 ≤ k ≤ 17, -21 ≤ l ≤ 21
Reflections collected	24124
Independent reflections	4800 [R_{int} = 0.0247, R_{sigma} = 0.0184]
Data/restraints/parameters	4800/2/372
Goodness-of-fit on F^2	1.028
Final R indexes [I>=2σ (I)]	R_1 = 0.0149, wR_2 = 0.0337
Final R indexes [all data]	R_1 = 0.0152, wR_2 = 0.0338
Largest diff. peak/hole / e Å^{-3}	0.22/-0.20
Flack parameter	0.201(16)

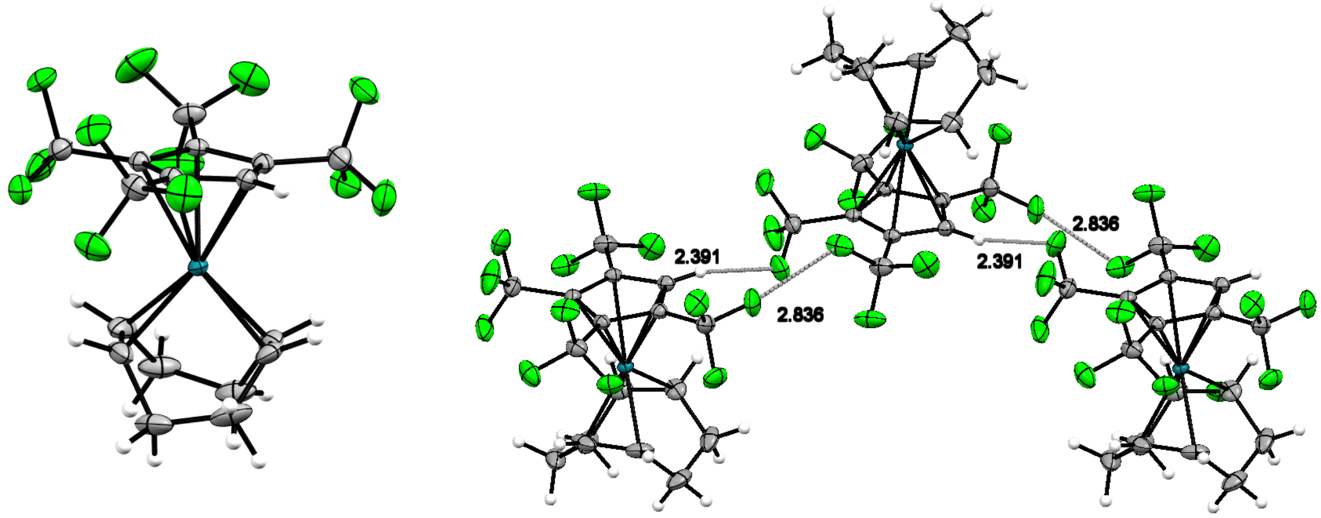

Figure 32. Molecular structure in solid state of [Rh(COD)($C_5(CF_3)_4H$)] (left) and its intermolecular arrangement (right). Ellipsoids are depicted with 50% probability level. Color code: white-hydrogen, grey-carbon, green-fluorine, blue-rhodium.

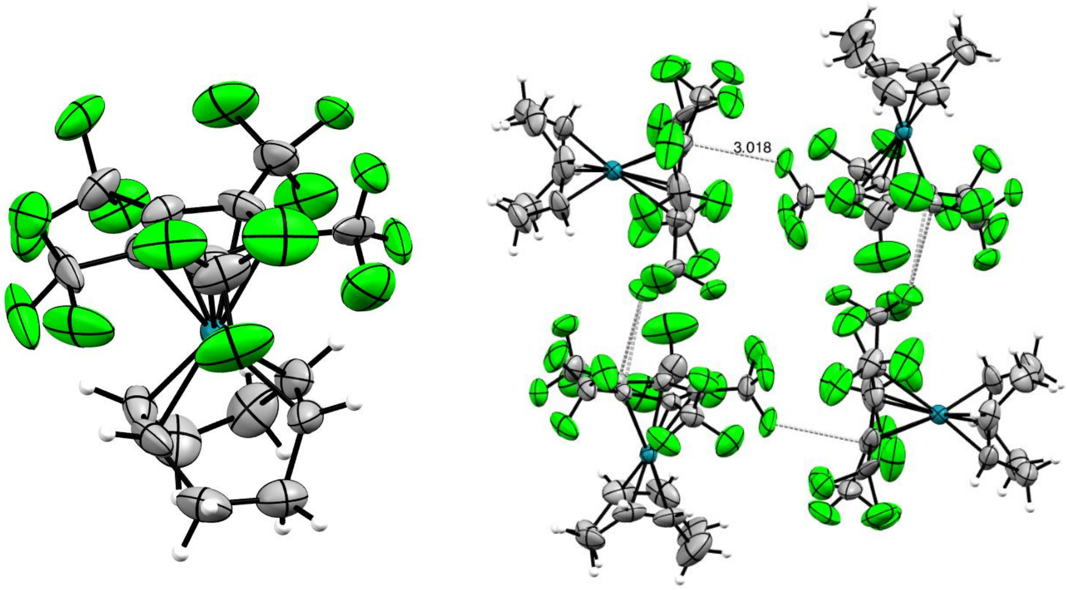

Figure 33. Molecular structure in solid state of [Rh(COD)($C_5(CF_3)_5$)] · 0.25 *n*-pentane (left) and its intermolecular arrangement (right). Disorder (rotation along C_{Cp}-CF_3, rotation of COD) and solvent molecules (0.25 *n*-pentane per [Rh(COD)($C_5(CF_3)_5$)]) are omitted for clarity. Ellipsoids are depicted with 50% probability level. Color code: white-hydrogen, grey-carbon, green-fluorine, blue-rhodium.

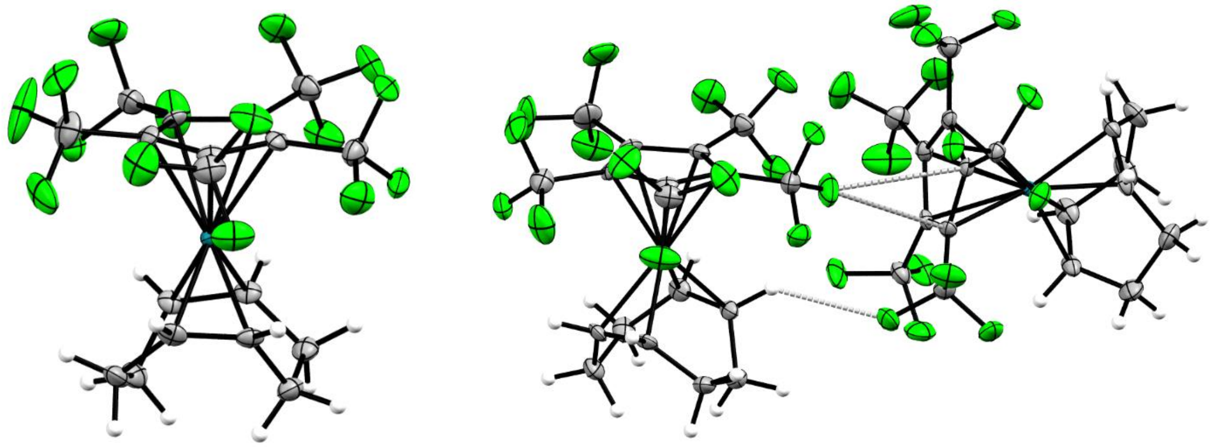

Figure 34. Molecular structure in solid state of [Rh(COD)($C_5(CF_3)_5$)] (left) and its intermolecular arrangement (right). Ellipsoids are depicted with 50% probability level. Color code: white-hydrogen, grey-carbon, green-fluorine, blue-rhodium.

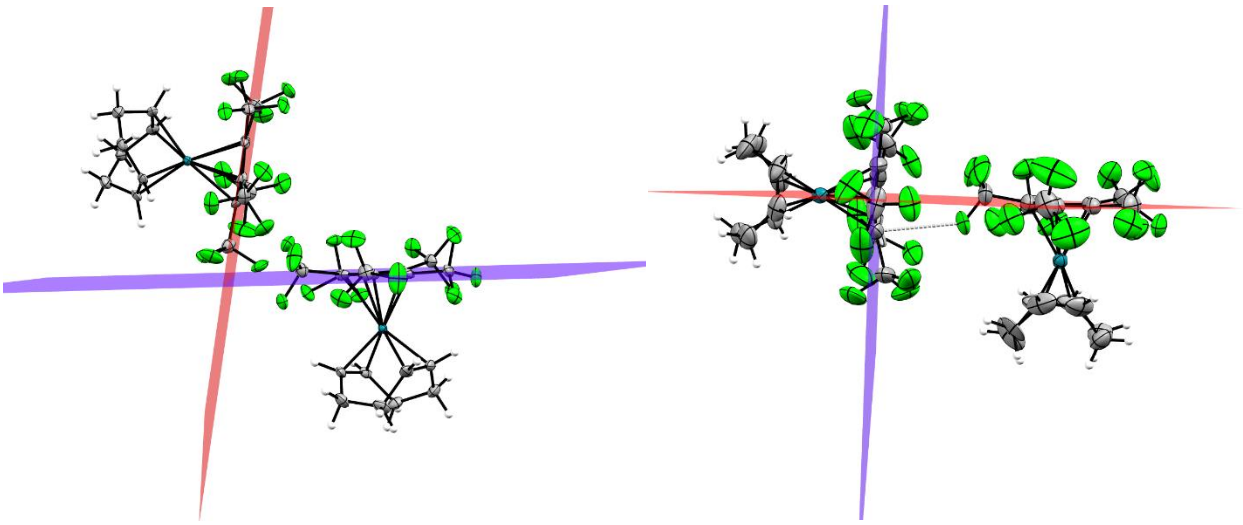

Figure 35. Packing details of the molecular structures in solid state of [Rh(COD)($C_5(CF_3)_5$)] from perfluorhexanes (left, angle between Cp planes 80.21°) and [Rh(COD)($C_5(CF_3)_5$)] · 0.25 *n*-pentane (right, angle between Cp planes 89.35°).

Table 5. Selected experimental bond lengths in Å of [Rh(COD)($C_5(CF_3)_4H$)].

Rh-C_{Cp}	C_{Cp}-C_{Cp}	C_{COD}=C_{COD}
2.241(3)	1.432(6)	1.400(6)
2.284(3)	1.427(5)	1.406(6)
2.226(3)	1.417(5)	
2.307(3)	1.419(6)	
2.301(3)	1.385(6)	

Table 6. Selected experimental bond lengths in Å of [Rh(COD)($C_5(CF_3)_5$)] (one asymmetric unit, with two molecules).

Molecule 1			Molecule 2		
Rh-C_{Cp}	C_{Cp}-C_{Cp}	C_{COD}=C_{COD}	Rh-C_{Cp}	C_{Cp}-C_{Cp}	C_{COD}=C_{COD}
2.323(4)	1.418(6)	1.405(7)	2.319(4)	1.427(5)	1.405(6)
2.224(4)	1.425(6)	1.402(7)	2.271(4)	1.416(7)	1.404(6)
2.346(4)	1.448(7)		2.293(4)	1.442(6)	
2.289(4)	1.407(5)		2.288(5)	1.399(6)	
2.264(4)	1.444(7)		2.227(4)	1.438(7)	

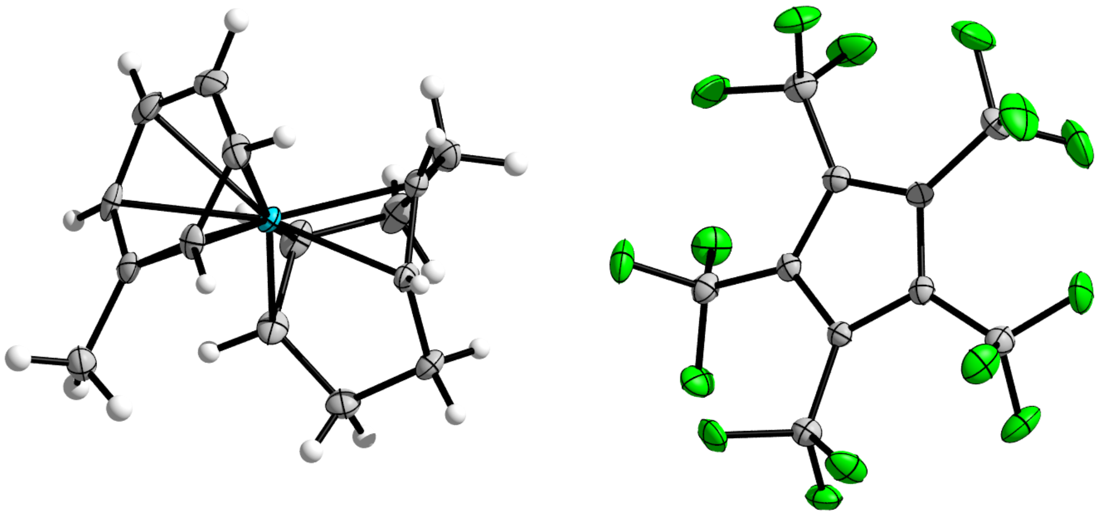

Figure 36. Molecular structure in solid state of [Rh(COD)(PhMe)][$C_5(CF_3)_5$]. Ellipsoids are depicted with 50% probability level. Color code: white-hydrogen, grey-carbon, green-fluorine, blue-rhodium.

DFT Calculations

[$C_5(CH_3)_5$]$^-$

monoanionic, diamagnetic, E = –390.2689532 Hartree

Table 7. Structure optimized coordinates (x,y,z) for [$C_5(CH_3)_5$]$^-$.

C1	2.2324060000	-1.5391410000	0.0048840000
C2	-0.7756910000	-2.5982640000	0.0046210000
C3	-0.9011140000	2.5573830000	0.0047740000
C4	2.1548690000	1.6458950000	0.0045560000
C5	0.9620550000	0.7353230000	-0.0056670000
C6	0.9966560000	-0.6877410000	-0.0056900000
C7	-2.7106170000	-0.0657930000	0.0046970000
C8	-0.3460610000	-1.1604250000	-0.0058000000
C9	-1.2104190000	-0.0294390000	-0.0055390000
C10	-0.4020050000	1.1421720000	-0.0056450000
H11	-0.0389480000	-3.2506570000	-0.4994610000
H12	-0.9139220000	-3.0239730000	1.0228820000
H13	-1.7394470000	-2.7435080000	-0.5178930000
H14	-3.1032300000	-0.9650500000	-0.5050680000
H15	-3.1583300000	-0.0725980000	1.0229250000
H16	-3.1466790000	0.8091530000	-0.5121640000
H17	-1.0559830000	2.9772700000	1.0230690000
H18	-0.1991570000	3.2444180000	-0.5030160000
H19	-1.8728520000	2.6545010000	-0.5140310000
H20	1.9429350000	2.6062720000	-0.5007410000
H21	2.5163680000	1.9102140000	1.0227490000
H22	3.0201700000	1.1960220000	-0.5167840000
H23	3.0745590000	-1.0482270000	-0.5173180000
H24	2.6067450000	-1.7845190000	1.0231630000
H25	2.0673070000	-2.5091450000	-0.4994620000

[C_5H_5]$^-$

monoanionic, diamagnetic, E = -193.6053669 Hartree

Table 8. Structure optimized coordinates (x,y,z) for [C_5H_5]$^-$.

C1	-1.1388780000	0.4011500000	0.0000730000
C2	-0.7334520000	-0.9591690000	0.0000470000
C3	0.6855820000	-0.9939530000	-0.0000760000
C4	1.1571600000	0.3448750000	0.0000490000
C5	0.0295880000	1.2070980000	-0.0000920000
H6	-1.3975910000	-1.8277020000	0.0004440000
H7	1.3063690000	-1.8939810000	-0.0001720000
H8	0.0563770000	2.3001260000	-0.0001450000
H9	-2.1701310000	0.7643940000	0.0004010000
H10	2.2049720000	0.6571610000	-0.0005270000

[C_5F_5]$^-$

monoanionic, diamagnetic, E = –689.9977389 Hartree

Table 9. Structure optimized coordinates (x,y,z) for [C_5F_5]$^-$.

F1	-0.8291340000	2.4406060000	-0.0000520000
F2	2.1053330000	-1.4871400000	-0.0001810000
F3	2.0649410000	1.5427350000	0.0002060000
F4	-2.5773660000	-0.0343640000	0.0002020000
F5	-0.7637740000	-2.4618400000	-0.0001740000
C6	0.9615700000	0.7184000000	-0.0000700000

C7	-0.3860980000	1.1365070000	-0.0005550000
C8	-1.2001890000	-0.0160010000	-0.0000730000
C9	-0.3556610000	-1.1463940000	0.0003520000
C10	0.9803800000	-0.6925090000	0.0003470000

$[C_5(CF_3)_4H]^-$

monoanionic, diamagnetic, E = −1542.4868912 Hartree

Table 10. Structure optimized coordinates (x,y,z) for $[C_5(CF_3)_4H]^-$.

F1	3.2115670000	-1.3255350000	-1.1705940000
F2	3.3265620000	-0.9696640000	0.9861950000
F3	2.5086520000	-2.8531470000	0.2239580000
F4	-3.2126700000	-1.3256430000	1.1699550000
F5	-3.3256790000	-0.9696180000	-0.9869710000
F6	-2.5085570000	-2.8531900000	-0.2238370000
F7	-1.7763750000	2.1761700000	-1.2334240000
F8	-2.8471320000	1.3493300000	0.4818850000
F9	-1.1138240000	2.6356610000	0.8018990000
F10	1.1136660000	2.6362420000	-0.8007290000
F11	1.7774060000	2.1749000000	1.2338880000
F12	2.8466940000	1.3491770000	-0.4829740000
C13	1.5897610000	1.6172530000	-0.0162210000
C14	2.5158130000	-1.4946670000	0.0082220000
C15	-2.5158960000	-1.4947710000	-0.0080650000
C16	-1.5898210000	1.6173370000	0.0160240000
C17	-0.7109930000	0.4194400000	0.0110240000
C18	0.7107010000	0.4195470000	-0.0113400000
C19	1.1415950000	-0.9419020000	-0.0015260000
C20	0.0000120000	-1.7626560000	0.0002940000
C21	-1.1416480000	-0.9420360000	0.0025470000
H22	0.0000740000	-2.8474360000	0.0009880000

$[C_5(CF_3)_5]^-$

monoanionic, diamagnetic, E = −1879.6769206 Hartree

Table 11. Structure optimized coordinates (x,y,z) for $[C_5(CF_3)_5]^-$.

F1	-2.1396480000	-2.5866320000	-1.0429150000
F2	-1.2462040000	-3.0603880000	0.9041830000
F3	-2.9107540000	-1.6747690000	0.7847480000
F4	-3.3366250000	0.3774830000	-0.8402830000
F5	-3.0815790000	0.9876830000	1.2542530000
F6	-2.5691000000	2.3532840000	-0.3575440000
F7	-0.3619850000	3.4112250000	0.8381680000
F8	1.5295120000	3.1425190000	-0.2140120000
F9	-0.3173580000	3.1547330000	-1.3435910000
F10	3.3029930000	1.0823270000	-0.8258880000
F11	2.8061490000	1.5170940000	1.2684370000
F12	3.3454820000	-0.4952870000	0.6853550000
F13	2.4586570000	-2.1140490000	-1.0889660000
F14	1.8654420000	-2.9032450000	0.8703500000
F15	0.6192220000	-3.2417930000	-0.8937090000
C16	1.3799740000	-2.2975370000	-0.2631080000
C17	-1.7539870000	-2.0314400000	0.1547740000
C18	0.2433950000	2.7018060000	-0.1714030000
C19	2.6334140000	0.5915790000	0.2692580000
C20	1.1889850000	0.2800840000	0.0237200000

C21	0.6272180000	-1.0261160000	-0.0191170000
C22	-2.4922850000	1.0461410000	0.0106020000
C23	-0.7873050000	-0.8974190000	0.0174930000
C24	-1.1043090000	0.4887300000	-0.0064250000
C25	0.1185960000	1.2188980000	-0.0136710000

$[C_5(CN)_5]^-$

monoanionic, diamagnetic, E = –655.1431712 Hartree

Table 12. Structure optimized coordinates (x,y,z) for $[C_5(CN)_5]^-$.

N1	-2.5145260000	-2.8386880000	0.0000310000
N2	1.9229750000	-3.2684650000	0.0001530000
N3	3.7028330000	0.8183820000	0.0001140000
N4	0.3658900000	3.7745550000	0.0000520000
N5	-3.4769200000	1.5140370000	0.0000960000
C6	2.5616230000	0.5663980000	-0.0000860000
C7	1.3302810000	-2.2612060000	-0.0001040000
C8	-1.7395410000	-1.9639000000	-0.0000220000
C9	0.2529080000	2.6113310000	-0.0000280000
C10	-2.4053940000	1.0474650000	-0.0000260000
C11	1.1834300000	0.2617090000	-0.0000630000
C12	0.6145470000	-1.0446600000	-0.0000770000
C13	-0.8036600000	-0.9073100000	-0.0000340000
C14	0.1168030000	1.2064330000	-0.0000410000
C15	-1.1112920000	0.4839510000	-0.0000390000

$[C_5Cl_5]^-$

monoanionic, diamagnetic, E = –2491.9644555 Hartree

Table 13. Structure optimized coordinates (x,y,z) for $[C_5Cl_5]^-$.

Cl1	-0.0000050000	2.9382040000	0.0000000000
Cl2	-2.7943330000	0.9080080000	0.0000000000
Cl3	-1.7269870000	-2.3770570000	0.0000000000
Cl4	1.7270040000	-2.3771000000	0.0000000000
Cl5	2.7943160000	0.9079530000	0.0000000000
C6	1.1435880000	0.3715670000	0.0000000000
C7	0.0000000000	1.2022870000	0.0000000000
C8	-1.1435280000	0.3715670000	0.0000000000
C9	0.7066880000	-0.9726920000	0.0000000000
C10	-0.7067330000	-0.9727480000	0.0000000000

$[Rh(COD)]^+$

monocationic, diamagnetic, E = –422.6603630 Hartree

Table 14. Structure optimized coordinates (x,y,z) for $[Rh(COD)]^+$.

Rh1	-1.1185780000	-0.0000100000	0.0000020000
C2	0.3216120000	-0.2159040000	-1.4865980000
C3	1.4898430000	-1.1058100000	-1.0866010000
C4	0.1942610000	1.1622450000	-1.0879710000
C5	1.1552270000	-1.9075190000	0.1897890000
C6	1.1551860000	1.9075430000	-0.1897880000
C7	1.4898330000	1.1058310000	1.0865900000
C8	0.3216160000	0.2159110000	1.4865980000
C9	0.1942860000	-1.1622400000	1.0879720000
H10	1.7167790000	1.7980480000	1.9073980000
H11	2.3853720000	0.4851920000	0.9431090000

H12	0.6861810000	2.8625670000	0.0855180000
H13	2.0697610000	2.1668730000	-0.7520400000
H14	-0.2239700000	-0.5166850000	-2.3911270000
H15	-0.4317920000	1.7992530000	-1.7307520000
H16	1.7167820000	-1.7980320000	-1.9074070000
H17	2.3853790000	-0.4851620000	-0.9431400000
H18	0.6862490000	-2.8625600000	-0.0855040000
H19	2.0698130000	-2.1668150000	0.7520370000
H20	-0.2239680000	0.5166870000	2.3911260000
H21	-0.4317600000	-1.7992580000	1.7307500000

[Rh(COD)($C_5(CH_3)_5$)]

neutral, diamagnetic, E = –813.2505509 Hartree

Table 15. Structure optimized coordinates (x,y,z) for [Rh(COD)($C_5(CH_3)_5$)].

Rh1	-0.1358790000	-0.0115830000	-0.0015800000
C2	1.7261460000	0.3522680000	2.7109550000
C3	1.8350340000	-2.4756810000	1.1972940000
C4	1.8523170000	-1.8627200000	-1.9532060000
C5	1.9374510000	1.2821760000	-2.3698020000
C6	1.8775710000	2.6668820000	0.5323550000
C7	1.7915630000	1.2035780000	0.2332740000
C8	1.8211960000	0.5922960000	-1.0474970000
C9	1.7594400000	-0.8484380000	-0.8563790000
C10	1.6996620000	0.1444060000	1.2284960000
C11	1.7500590000	-1.1272890000	0.5527830000
C12	-1.6548810000	-0.1441660000	1.4780100000
C13	-2.8405760000	-1.0234070000	1.0829040000
C14	-1.5113470000	1.2101220000	1.0387310000
C15	-2.5203650000	-1.8748820000	-0.1606130000
C16	-2.4710380000	1.9198760000	0.0993780000
C17	-2.7855720000	1.0786650000	-1.1531780000
C18	-1.6183160000	0.1633050000	-1.5213710000
C19	-1.5218040000	-1.1936770000	-1.0804460000
H20	1.2693390000	2.9354130000	1.4080800000
H21	2.9157210000	2.9711470000	0.7509490000
H22	1.5246230000	3.2712240000	-0.3145530000
H23	1.5791120000	2.3191890000	-2.3146300000
H24	2.9843110000	1.3079550000	-2.7182600000
H25	1.3505440000	0.7681770000	-3.1448480000
H26	1.3290870000	-1.5272200000	-2.8601150000
H27	2.9032840000	-2.0498610000	-2.2319330000
H28	1.4144630000	-2.8236200000	-1.6498220000
H29	2.7600640000	0.4641980000	3.0791030000
H30	1.1756400000	1.2583130000	3.0021640000
H31	1.2765570000	-0.4974200000	3.2429340000
H32	1.3733750000	-3.2497140000	0.5685100000
H33	2.8829880000	-2.7751350000	1.3756840000
H34	1.3192610000	-2.4895700000	2.1676140000
H35	-3.0101480000	1.7448300000	-2.0000790000
H36	-3.6936900000	0.4753140000	-0.9983460000
H37	-1.9881000000	2.8589740000	-0.2141460000
H38	-3.4008270000	2.2141750000	0.6258040000
H39	-1.1927150000	-0.3945670000	2.4385670000
H40	-0.9646660000	1.8852330000	1.7087870000

H41	-3.1065640000	-1.6808730000	1.9245870000
H42	-3.7248760000	-0.3919840000	0.9047880000
H43	-2.0688780000	-2.8250880000	0.1657050000
H44	-3.4457400000	-2.1456130000	-0.7069620000
H45	-1.1249180000	0.4020950000	-2.4700670000
H46	-0.9819070000	-1.8842310000	-1.7385630000

[Rh(COD)(C_5H_5)]

neutral, diamagnetic, E = –616.5705928 Hartree

Table 16. Structure optimized coordinates (x,y,z) for [Rh(COD)(C_5H_5)].

Rh1	-0.3902400000	-0.0017130000	0.0010200000
C2	-2.3289070000	1.2276330000	-0.0141290000
C3	-2.3562070000	0.4043420000	1.1377130000
C4	-2.3375170000	-0.9735530000	0.7076680000
C5	-2.2457380000	0.3580770000	-1.1630840000
C6	-2.3202860000	-1.0055510000	-0.7130770000
C7	1.1081550000	-0.1639400000	-1.4913280000
C8	2.2857450000	-1.0581040000	-1.1079730000
C9	0.9881610000	1.1950000000	-1.0594580000
C10	1.9713880000	-1.8955540000	0.1460420000
C11	1.9700310000	1.8964750000	-0.1385170000
C12	2.2901740000	1.0574840000	1.1129450000
C13	1.1149150000	0.1627540000	1.5019510000
C14	0.9943680000	-1.1942530000	1.0728770000
H15	-2.3374270000	2.3134690000	-0.0314890000
H16	-2.3864750000	0.7445000000	2.1694920000
H17	-2.3604690000	-1.8384950000	1.3657740000
H18	-2.2244040000	0.6825560000	-2.2008730000
H19	-2.3287770000	-1.8917040000	-1.3403130000
H20	2.5362510000	1.7234760000	1.9534970000
H21	3.1851670000	0.4377640000	0.9482510000
H22	1.5063700000	2.8441690000	0.1762920000
H23	2.8945840000	2.1717320000	-0.6832080000
H24	0.6283520000	-0.4141910000	-2.4428330000
H25	0.4422530000	1.8718530000	-1.7266000000
H26	2.5272940000	-1.7253850000	-1.9488130000
H27	3.1816050000	-0.4381600000	-0.9486620000
H28	1.5059810000	-2.8432940000	-0.1658910000
H29	2.8987220000	-2.1705920000	0.6859590000
H30	0.6353610000	0.4156480000	2.4525130000
H31	0.4507070000	-1.8711520000	1.7411920000

[Rh(COD)(C_5F_5)]

neutral, diamagnetic, E = –1112.9339632 Hartree

Table 17. Structure optimized coordinates (x,y,z) for [Rh(COD)(C_5F_5)].

Rh1	0.1416320000	-0.0238050000	-0.0071370000
F2	-1.9564660000	2.4532400000	-0.6668740000
F3	-1.9839900000	1.3587820000	2.1427620000
F4	-1.8382810000	-1.6124960000	1.9691060000
F5	-1.8089140000	0.1417340000	-2.5496900000
F6	-1.9140810000	-2.4059190000	-0.9417000000
C7	-1.8218570000	1.1734070000	-0.3016570000
C8	-1.8334690000	0.6636150000	1.0097810000
C9	-1.6959270000	-0.7825450000	0.9231850000

C10	-1.6774150000	0.0484890000	-1.2160370000
C11	-1.7111600000	-1.1694420000	-0.4582000000
C12	1.6882320000	-0.1326720000	-1.4938030000
C13	2.8715830000	-1.0062920000	-1.0879160000
C14	1.5289010000	1.2157900000	-1.0653050000
C15	2.5520160000	-1.8591700000	0.1550340000
C16	2.4617220000	1.9433420000	-0.1157060000
C17	2.7987770000	1.1065230000	1.1330850000
C18	1.6522840000	0.1749800000	1.5163920000
C19	1.5672950000	-1.1797630000	1.0870260000
H20	3.0188910000	1.7764010000	1.9772990000
H21	3.7126100000	0.5148840000	0.9716480000
H22	1.9545380000	2.8678140000	0.2000580000
H23	3.3821580000	2.2632490000	-0.6415910000
H24	1.2090170000	-0.4030430000	-2.4389210000
H25	0.9563160000	1.8742020000	-1.7278650000
H26	3.1405290000	-1.6644630000	-1.9270830000
H27	3.7509750000	-0.3696200000	-0.9085710000
H28	2.0966140000	-2.8071920000	-0.1691360000
H29	3.4775760000	-2.1317060000	0.6979460000
H30	1.1408430000	0.4215280000	2.4517120000
H31	1.0162080000	-1.8664970000	1.7379840000

[Rh(COD)($C_5(CF_3)_4H$)]

neutral, diamagnetic, E = −1965.3664412 Hartree

Table 18. Structure optimized coordinates (x,y,z) for [Rh(COD)($C_5(CF_3)_4H$)].

Rh1	-0.4895960000	-0.7021400000	-0.0188580000
F2	3.6444910000	0.1618570000	-0.6215280000
F3	2.7675070000	1.9401280000	-1.5296300000
F4	2.2139850000	-0.0479430000	-2.2565150000
F5	3.4941880000	-0.2861380000	2.1905060000
F6	2.8208170000	-1.9127960000	0.8876330000
F7	1.8574440000	-1.5923970000	2.8219210000
F8	0.1587960000	1.9508320000	-2.5063460000
F9	0.3338920000	3.6005910000	-1.0787560000
F10	-1.5755070000	2.5689670000	-1.3343720000
F11	-2.8911070000	1.6959040000	0.9482620000
F12	-1.6052200000	3.3788080000	1.4901710000
F13	-1.9404930000	1.7442610000	2.9127900000
C14	-0.9518000000	-1.2086890000	-2.0323290000
C15	-2.1524590000	-0.8662650000	-1.3475300000
C16	-3.1981740000	-1.8867240000	-0.9123130000
C17	-2.9744370000	-2.3327490000	0.5437980000
C18	-0.5087960000	-2.6169210000	-2.3698720000
C19	-1.5093750000	-2.3093600000	0.9301810000
C20	-0.6492620000	-3.5728710000	-1.1725350000
C21	-0.4448930000	-2.8382300000	0.1476420000
C22	1.3833790000	0.6492710000	-0.1158370000
C23	1.3803230000	-0.0165290000	1.1452300000
C24	0.1477710000	1.4157030000	-0.1868250000
C25	-0.5251370000	1.3082840000	1.0918950000
C26	0.2154520000	0.3954160000	1.8761040000
C27	-1.7427250000	2.0298260000	1.5947080000
C28	-0.2311730000	2.3820540000	-1.2792330000

C29	2.4970820000	0.6670940000	-1.1334240000
C30	2.3976180000	-0.9508400000	1.7436910000
H31	-0.5158140000	-0.4355980000	-2.6713000000
H32	-2.5280190000	0.1486880000	-1.5012140000
H33	-1.0596850000	-2.9923500000	-3.2530260000
H34	0.5489790000	-2.5650520000	-2.6665660000
H35	-1.6341730000	-4.0629580000	-1.1717090000
H36	0.0928340000	-4.3799410000	-1.2536350000
H37	-1.3288770000	-2.2335890000	2.0079940000
H38	0.4713890000	-3.0955880000	0.6855560000
H39	-3.4058370000	-3.3347360000	0.7285480000
H40	-3.5045030000	-1.6413230000	1.2155980000
H41	-3.1762190000	-2.7487760000	-1.5952350000
H42	-4.1985970000	-1.4422930000	-1.0126320000
H43	-0.0391580000	0.0703570000	2.8788760000

[Rh(COD)($C_5(CF_3)_5$)]

neutral, diamagnetic, E = −2302.5434740 Hartree

Table 19. Structure optimized coordinates (x,y,z) for [Rh(COD)($C_5(CF_3)_5$)].

Rh1	-0.4199860000	-0.5695670000	-0.6284370000
F2	3.2065850000	-0.3369380000	-1.3079710000
F3	3.4267480000	1.6805840000	-0.4810310000
F4	3.7976890000	-0.0451410000	0.7726910000
F5	2.3879300000	-2.2205450000	1.1461610000
F6	2.4718050000	-0.9504220000	2.9400060000
F7	0.7962320000	-2.2969130000	2.6196860000
F8	-1.5937090000	-1.6536430000	2.5131700000
F9	-0.9582010000	0.0290940000	3.7589790000
F10	-2.6238250000	0.2342440000	2.3464580000
F11	1.5707860000	1.8802900000	-2.3104100000
F12	-0.4064610000	2.7093040000	-1.9811550000
F13	1.3153600000	3.5724940000	-0.9421770000
F14	-2.8663110000	2.0675280000	0.2787730000
F15	-1.8531690000	2.6713760000	2.1314700000
F16	-1.2919300000	3.5802030000	0.2478770000
C17	0.7593390000	2.4096790000	-1.3533100000
C18	-1.6638100000	2.4055910000	0.8102880000
C19	-1.3962580000	-0.3106770000	2.5191760000
C20	1.6433490000	-1.4562110000	1.9883440000
C21	2.9906090000	0.4257580000	-0.2036080000
C22	1.5342730000	0.4540780000	0.2334910000
C23	0.9348180000	-0.3412890000	1.2447060000
C24	-0.4186790000	0.1605990000	1.4559850000
C25	0.5540070000	1.4278940000	-0.2147690000
C26	-0.6001810000	1.3397420000	0.6334710000
C27	-0.8991020000	-0.4895060000	-2.7298640000
C28	-2.2418410000	-1.1149690000	-3.0841360000
C29	0.2906900000	-1.2368020000	-2.5371190000
C30	-3.0903230000	-1.3627330000	-1.8260100000
C31	0.3993540000	-2.7460300000	-2.5802140000
C32	-0.7167760000	-3.4338170000	-1.7749960000
C33	-1.1453250000	-2.5835820000	-0.5847130000
C34	-2.2393100000	-1.6726090000	-0.6138610000
H35	-0.3630480000	-4.4090580000	-1.4115030000

H36	-1.5903090000	-3.6426610000	-2.4101550000
H37	1.3740430000	-3.0178120000	-2.1491120000
H38	0.4170830000	-3.0984360000	-3.6287430000
H39	-0.7758030000	0.5520010000	-3.0338690000
H40	1.2308690000	-0.7096120000	-2.7192420000
H41	-2.7807870000	-0.4443240000	-3.7683330000
H42	-2.0763010000	-2.0502490000	-3.6387450000
H43	-3.6701350000	-0.4565700000	-1.5968770000
H44	-3.8294310000	-2.1695800000	-1.9893580000
H45	-0.8812830000	-2.9858730000	0.3970000000
H46	-2.7402990000	-1.4816410000	0.3386980000

[Rh(COD)($C_5(CN)_5$)]

neutral, diamagnetic, E = −1077.9758230 Hartree

Table 20. Structure optimized coordinates (x,y,z) for [Rh(COD)($C_5(CN)_5$)].

Rh1	-0.3178950000	-0.0254820000	-0.0220320000
N2	2.0116000000	-3.0250190000	-2.0289950000
N3	1.8345460000	1.1235270000	-3.5561060000
N4	1.3982470000	3.9052000000	-0.0214750000
N5	1.1079520000	1.3807860000	3.6433050000
N6	1.7460150000	-2.8903350000	2.4724060000
C7	1.4752970000	2.7416150000	0.0023890000
C8	1.7949230000	0.8252380000	-2.4297110000
C9	1.9183170000	-2.0728640000	-1.3629230000
C10	1.2839380000	0.9864690000	2.5608430000
C11	1.7100030000	-1.9838000000	1.7392250000
C12	1.5519630000	1.3318700000	0.0454560000
C13	1.7132850000	0.4489280000	-1.0690590000
C14	1.7691640000	-0.9131360000	-0.5664940000
C15	1.4465690000	0.5060630000	1.2380840000
C16	1.6556160000	-0.8797060000	0.8608050000
C17	-1.9050320000	-0.2675310000	1.3807130000
C18	-2.9876830000	-1.2391370000	0.9262650000
C19	-1.8292060000	1.0926770000	0.9633310000
C20	-2.5283600000	-2.0654330000	-0.2895080000
C21	-2.7474200000	1.7596580000	-0.0378820000
C22	-2.9810710000	0.8892910000	-1.2865180000
C23	-1.7540190000	0.0467220000	-1.6120380000
C24	-1.5767370000	-1.2965130000	-1.1823610000
H25	-3.2122620000	1.5329770000	-2.1465370000
H26	-3.8514170000	0.2311770000	-1.1519510000
H27	-2.2745840000	2.7063100000	-0.3388450000
H28	-3.7024340000	2.0352070000	0.4466030000
H29	-1.4303330000	-0.5050420000	2.3384230000
H30	-1.3260590000	1.7841430000	1.6491880000
H31	-3.2354630000	-1.9136710000	1.7574550000
H32	-3.9068390000	-0.6800480000	0.6992350000
H33	-1.9982060000	-2.9624100000	0.0627340000
H34	-3.3910740000	-2.4303200000	-0.8768580000
H35	-1.1949250000	0.3513200000	-2.5031310000
H36	-0.9169170000	-1.9245260000	-1.7925300000

[Rh(COD)(C_5Cl_5)]

neutral, diamagnetic, E = –2914.8747275 Hartree

Table 21. Structure optimized coordinates (x,y,z) for [Rh(COD)(C_5Cl_5)].

Rh1	0.5073780000	-0.0187420000	-0.0137000000
Cl2	-1.6646470000	0.8738460000	-2.7502170000
Cl3	-1.4869030000	2.9839840000	-0.0154490000
Cl4	-1.2994560000	1.0119250000	2.8055870000
Cl5	-1.6333590000	-2.2780630000	1.8450920000
Cl6	-1.7439380000	-2.3472840000	-1.6079890000
C7	-1.3622960000	1.2749640000	0.0029090000
C8	-1.4542110000	0.4047690000	-1.1158400000
C9	-1.2513580000	0.4388420000	1.1846670000
C10	1.9587220000	1.1205710000	1.0205740000
C11	2.9018480000	1.8197880000	0.0605520000
C12	3.1743280000	0.9827380000	-1.2041340000
C13	1.9703860000	0.1201090000	-1.5711330000
C14	-1.4173010000	-0.9454410000	0.7899400000
C15	-1.4845800000	-0.9649100000	-0.6294620000
C16	2.0582410000	-0.2455640000	1.4240680000
C17	1.8227220000	-1.2349530000	-1.1625210000
C18	2.7911160000	-1.9866250000	-0.2683070000
C19	3.1857350000	-1.1741500000	0.9804320000
H20	1.4294240000	1.7935060000	1.7048640000
H21	3.8457580000	2.0951760000	0.5691390000
H22	2.4246630000	2.7672630000	-0.2333610000
H23	4.0575280000	0.3412330000	-1.0662730000
H24	3.4125810000	1.6503490000	-2.0451040000
H25	1.4327480000	0.4194940000	-2.4769310000
H26	1.5806980000	-0.5059510000	2.3741350000
H27	4.0944050000	-0.5829450000	0.7915550000
H28	3.4359090000	-1.8600360000	1.8029610000
H29	3.6860970000	-2.3023830000	-0.8379880000
H30	2.2890590000	-2.9123260000	0.0515240000
H31	1.2001670000	-1.8757480000	-1.7978910000

Table 22. Selected MO-energies in eV of $[C_5H_5]^-$, $[C_5F_5]^-$, $[C_5(CF_3)_5]^-$.

MO	E($[C_5H_5]^-$)	E($[C_5F_5]^-$)	E($[C_5(CF_3)_5]^-$)
HOMO–1	**–2.71** (σ-don.)	**–3.03** (σ-don.)	**–5.81** (σ-don.)
HOMO	**+0.97, +0.97** (π-don.)	**+0.30, +0.30** (π-don.)	**–2.66, –2.65** (π-don.)
LUMO	+5.64	+3.14	**+1.88, +1.92** (δ-acc.)
LUMO+1	+6.41, +6.41	+6.20, +6.20	+4.18, +4.19
LUMO+2	**+6.43, +6.43** (δ-acc.)	**+6.22, +6.22** (δ-acc.)	+4.95

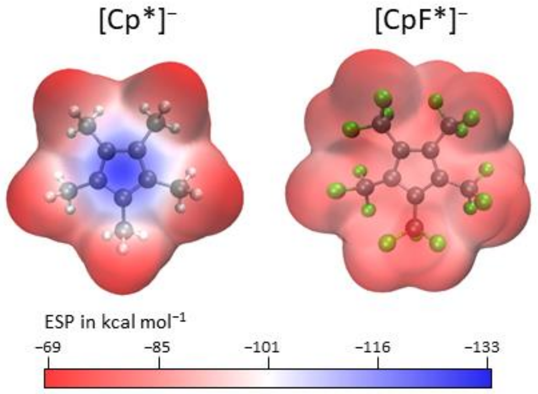

Figure 37. Electrostatic surface potential of $[C_5(CH_3)_5]^-$ (left) and $[C_5(CF_3)_5]^-$ (right).

References

[1] H. E. Gottlieb, V. Kotlyar, A. Nudelman, *J. Org. Chem.* **1997**, *62*, 7512–7515.

[2] G. R. Fulmer, A. J. M. Miller, N. H. Sherden, H. E. Gottlieb, B. M. Stoltz, J. E. Bercaw, K. I. Goldberg, *Organometallics* **2010**, *29*, 2176–2179.

[3] M. R. Willcott, *J. Am. Chem. Soc.* **2009**, *131*, 13180.

[4] O. V. Dolomanov, L. J. Bourhis, R. J. Gildea, J. A. K. Howard, H. Puschmann, *J. Appl. Cryst.* **2009**, *42*, 339–341.

[5] G. M. Sheldrick, *Acta Cryst.* **2015**, *A71*, 3–8.

[6] G. M. Sheldrick, *SHELXL Version 2014/7, Program for Crystal Structure Solution and Refinement*; Göttingen, Germany, 2014.

[7] G. M. Sheldrick, *Acta Cryst.* **2008**, *A64*, 112–122.

[8] K. Brandenburg, Diamond: Crystal and Molecular Structure Visualization http://www.crystalimpact.com/diamond.

[9] Persistence of Vision Pty. Ltd. Persistence of Vision Raytracer. Ltd., Persistence of Vision Pty. 2004.

[10] Gaussian 16, Revision C.01, M. J. Frisch, G. W. Trucks, H. B. Schlegel, G. E. Scuseria, M. A. Robb, J. R. Cheeseman, G. Scalmani, V. Barone, G. A. Petersson, H. Nakatsuji, X. Li, M. Caricato, A. V. Marenich, J. Bloino, B. G. Janesko, R. Gomperts, B. Mennucci, H. P. Hratchian, J. V. Ortiz, A. F. Izmaylov, J. L. Sonnenberg, D. Williams-Young, F. Ding, F. Lipparini, F. Egidi, J. Goings, B. Peng, A. Petrone, T. Henderson, D. Ranasinghe, V. G. Zakrzewski, J. Gao, N. Rega, G. Zheng, W. Liang, M. Hada, M. Ehara, K. Toyota, R. Fukuda, J. Hasegawa, M. Ishida, T. Nakajima, Y. Honda, O. Kitao, H. Nakai, T. Vreven, K. Throssell, J. A. Montgomery, Jr., J. E. Peralta, F. Ogliaro, M. J. Bearpark, J. J. Heyd, E. N. Brothers, K. N. Kudin, V. N. Staroverov, T. A. Keith, R. Kobayashi, J. Normand, K. Raghavachari, A. P. Rendell, J. C. Burant, S. S. Iyengar, J. Tomasi, M. Cossi, J. M. Millam, M. Klene, C. Adamo, R. Cammi, J. W. Ochterski, R. L. Martin, K. Morokuma, O. Farkas, J. B. Foresman, and D. J. Fox, Gaussian, Inc., Wallingford CT, 2016.

[11] M. D. Hanwell, D. E. Curtis, D. C. Lonie, T. Vandermeersch, E. Zurek, G. R. Hutchison, *J. Cheminformatics* **2012**, *4*, 17.

[12] G. A. Zhurko, in *ChemCraft*, http://www.chemcraftprog.com.

[13] T. Lu, F. Chen, *J. Comput. Chem.* **2012**, *33*, 580-592.

[14] W. Humphrey, A. Dalke, K. Schulten, *J. Molec. Graphics* **1996**, *14*, 33-38.

[15] ^{1}H- and ^{13}C{^{1}H}-NMR ethyl signals of $[NEt_4][C_5(CF_3)_4H]$ are superimposed with those of $[NEt_4][C_5(CF_3)_5]$. ^{13}C{^{19}F}-NMR Cp signals of $[NEt_4][C_5(CF_3)_4H]$ are invisible, due to minor substrate amounts and pronounced coupling patterns.

[16] E. P. Janulis, A. J. Arduengo, *J. Am. Chem. Soc.* **1983**, *105*, 3563–3567.

[17] R. D. Chambers, W. K. Gray, J. F. S. Vaughan, S. R. Korn, M. Médebielle, A. S. Batsanov, C. W. Lehmann, J. A. K. Howard, *Perkin Trans.* **1997**, 135–146.
